# The replicability of real-world evidence for the surgical treatment of proximal humeral fractures in older patients evaluated using sceptical p-value

**DOI:** 10.1038/s41598-026-70157-6

**Published:** 2026-09-08

**Authors:** Jeanette Koeppe, Karen Fischhuber, Josef Stolberg-Stolberg, Ursula Marschall, Michael J. Raschke, Andreas Faldum, J. Christoph Katthagen

**Affiliations:** 1 Department of Epidemiology and Biostatistics, Public and Global Health, Medical University Lausitz – Carl Thiem, Thiemstr. 111, Cottbus, 03048 Germany; 2 Institute of Biostatistics and Clinical Research, University of Münster, Schmeddingstr. 56, Münster, 48149 Germany; 3 Research Group Mathematical Surgery, University of Münster, University Hospital Münster, Albert-Schweitzer-Campus 1, Building W1, Münster, 48149 Germany; 4 Department of Trauma-, Hand- and Reconstructive Surgery, University Hospital Münster, Albert-Schweitzer-Campus 1, Building W1, Münster, 48149 Germany; 5 BARMER Institute for Health System Research, BARMER, Lichtscheider Str. 89, Wuppertal, 42285 Germany

**Keywords:** Real-world evidence, Replication, Sceptical p-value, Health service research, Proximal humeral fracture, Reverse total shoulder arthroplasty, Fracture repair, Epidemiology, Outcomes research

## Abstract

The replicability of study results plays a central role, particularly in the context of real-world evidence (RWE). However, the evaluation of the agreement between two study results is not straightforward and different metrics are available. As an example for RWE, the surgical treatment of proximal humeral fractures in older patients using locked plate fixation (LPF) or reverse total shoulder arthroplasty (RTSA) was analyzed by two different studies based on the data of the largest German health insurance company, but the generalizability of the results should be further addressed. Independent data of a different German insurance company were used to emulate two existing studies evaluating different short- and long-term safety endpoints after surgical treatment of proximal humeral fracture in older patients using RTSA vs LPF. Study protocol was a priori registered. The sceptical p-value was used to judge the agreement between known original effect estimate (OES) and the observed emulated treatment effect estimate (EES) and compared to other existing binary agreement metrics. Overall, 7 (64%) of the 11 evaluated endpoints were successfully replicated. The Pearson correlation coefficient ($r_p$ = 0.97) and the intra-class correlation coefficient (ICC3 = 0.94) indicated a good, positive correlation between original and emulation study. OES was successfully replicated for all short-term endpoints and surgical complications during follow-up (all $p_\text {scep} < 0.025$). Furthermore, the treatment effect for surgical complications during follow-up was successfully replicated using an independent data base. The sceptical p-value was a useful and well-interpretable metric to evaluate the agreement between original and emulation study.

Trial registration: A priori registered at ClinicalTrials.gov (Identifier: NCT06537024).

## Introduction

Randomized controlled clinical trials (RCTs) are the gold standard for proving the efficacy of a treatment, but external validity can be limited, particularly for highly controlled trials with typically small and highly selected patient cohorts^3–6^. To prove the efficacy of a new treatment, a study environment is established that can be compared to a clean laboratory setting and is idealized to the highest degree for the cause of bias reduction. However, the high standardization of treatment and the strict limitation of the patient population imply that the study environment does not always reflect the real situation in the healthcare system. Pragmatic RCTs are designed to better reflect routine clinical practice. Nevertheless, even pragmatic trials may still differ from everyday care due to protocol-driven monitoring and follow-up, site selection, and differences in adherence and provider expertise^7–10^. Therefore, complementary analyses using real-world data (RWD; including conceptual replication across independent data sources) can further inform generalizability and strengthen confidence in findings. Thus, due to a higher variability, there is evidence that drugs (or treatment in general) perform worse in daily clinical care than in RCTs, which was known in the literature as efficacy-effectiveness gap^4,11^. However, systematic analyzes failed to prove general significant differences between the effect estimate in observational studies and RCTs^12–16^. Even though it is necessary to prove the efficacy of a (new) therapy using RCTs, the potential lack of external validity of RCTs implies that the findings are not necessarily generalizable to all patients in daily clinical healthcare, making post-market analysis of treatments in actual real-world care necessary to ensure patient safety^4,17,18^. Furthermore, studies based on so-called real-world data play an increasingly important role in regulatory decisions^18–20^ and complement the information provided by RCTs. RWD have a great potential to create real-world evidence (RWE) due to growing availability of new data sources, especially for research questions that are difficult to address otherwise, e.g. for rare diseases or patients with comorbidities, often defined as exclusion criteria^18,21–23^.

Current use cases evaluated the surgical treatment of proximal humeral fracture (PHF) in the older people using German statutory health insurance data^1,2,24–26,63^. Due to an aging society, managing osteoporosis-related fractures is a growing global challenge^27^. They were responsible for the loss of annually more than 37 billion EUR and more than one million quality-adjusted years in Europe^28^. With an incidence in older patients of approx. 290 – 354/100,000 population-years^29,30^, the PHF is the third-most common fracture in older patients. Along with a fracture fixation using locked plate fixation (LPF), the reverse total shoulder arthroplasty (RTSA) is one of the most common surgical treatment options for older patients and has been increasingly used over the last decade^1^. However, even among highly specialist surgeons, a consensus about the optimal treatment of PHF is still missing, also caused by the small number of RCTs with contradictory results^31–35^. Based on German health claims data of more than 50,000 patients, it was observed that RTSA compared to LPF was associated with higher complications during short-term^1^, but had improved outcome on long-term^2^. These findings highlight the utility of RWD in assessing treatment safety and effectiveness and underscore the importance of tailoring treatment to individual risk profiles.

However, in the case of studies based on insurance data, the generalizability of the results to the entire population is sometimes questioned, especially when the insurance holders differ socio-economically between different companies. It is hence important to check the replicability of the results in order to strengthen their credibility, but remains a challenge due to unique data structures^21^.

In general, there is a distinction between direct and conceptual replication. A direct replication (also named reproduction) is defined as an evaluation of an independent investigators to obtain the same results when applying the same design and operational choices at the same data source (e.g. same insurance company, but with new data collection)^36,37^. As an example, Wang et al.^38^ directly replicated 150 RWD studies using the same database and the methods described in the original paper to emulate the design. Even though the majority of the results were replicated, there are subgroups in which the results could not be reproduced, which was explained by incomplete or non-transparent reporting and updated data^38^. The analysis showed impressively the importance of transparent reporting of the methodology in the context of RWE. In contrast, a conceptual replication is defined as an evaluation of an independent investigators to obtain the similar findings when applying the same design and operational choices at different/ independent data sources (e.g. different insurance company)^36^. As an example for conceptual replications, target trial emulations have become increasingly important in recent years. As in the original formulation, a target trial emulation is an emulation of a “hypothetical” RCT, using a two-step process^39,40^. In the first step, the causal questions are defined in a protocol for the hypothetical RCT, which has to specify the key elements to define the causal estimands (i.e. eligibility criteria, treatment strategies, treatment assignment, the start and end of follow-up, outcomes, causal contrasts) and the data analysis plan^39^. The RCT described in the protocol is defined as the target study for causal inference, which will be emulated using observational data in the second step^39,40^. Emulations of RCTs using RWD are used for regulatory decision making, but are also used to evaluate, why an emulation of RCTs in daily healthcare settings leads to different results^18–20,41–43^. As one example, the RCT DUPLICATE initiative (Randomized, Controlled Trials Duplicated Using Prospective Longitudinal Insurance Claims: Applying Techniques of Epidemiology) conducted the emulation of 32 RCTs (conceptual replications) with RWD to check, if and under which circumstances RWD studies explicitly designed to emulate RCTs are useful to draw the same causal conclusions^41,42,44^.

Whether direct or conceptual, the evaluation of the agreement between two study results (i.e. two treatment effects) is not straightforward and different metrics are available, e.g. checking regulatory and estimated agreement or determining the sceptical p-value^41,44–48^. However, even though different methods are available, an established standard method to evaluate the replicability is missing today^46^, but would further improve the evidence of conceptual replications, especially in the case of target trials emulations of an existing RCT or RWE study.

In the study at hand, the findings for safety after surgical treatment of proximal humeral fractures in older patients, as observed in Koeppe et al.^1^ and Stolberg-Stolberg et al.^2^, were conceptually replicated using an independent database from a different insurance company within the same healthcare system. The agreement of the study results was assessed using sceptical p-value and compared to various binary metrics. The aim of the study is to evaluate the replicability of these findings and to investigate how the sceptical p-value performs in judging the agreement in the context of RWE.

## Methods

### Emulation study

#### Data cohort of the emulation study

Study design and statistical methods were pre-defined and a priori registered at ClinicalTrials.gov (Identifier: NCT06537024; see study protocol) before the first effect size was calculated.

The original studies by Koeppe et al.^1^ and Stolberg-Stolberg et al.^2^ were based on data from the Federal Association of Local Health Insurance Funds (AOK). Retrospective health claims data (in- and outpatient data) of the German BARMER health insurance from 2005 to 2022 were available for the emulation study. Diagnoses were defined based on International Statistical Classification of Diseases, German Modification (ICD-10 GM) and procedures were coded using German procedure classification (*Operationen- und Prozedurenschlüssel* – OPS). The study cohort was defined in accordance to Koeppe et al.^1^, to establish an emulation of the study design presented there. Thus, all older patients (age *≥ * 65 years) with inpatient coded LPF (OPS 5-794.21, 5-794.k1) or RTSA (OPS 5-824.21) and with inpatient coded PHF (ICD-10 GM S42.2) from 01/2011 to 09/2022 were included to the study. Note, as in the original studies, our analysis was restricted to RTSA versus LPF and did not assess other fixation methods or anatomic total shoulder arthroplasty. The first hospitalization was defined as the index case. Exclusion criteria were defined in accordance to Koeppe et al.^1^ and Stolberg-Stolberg et al.^2^ (see CONSORT statement in Appendix Figure A1). However, a COVID-19 infection during hospitalization was additionally defined as an exclusion criteria to avoid bias. This exclusion was intended to reduce misattribution of acute COVID-19-related perioperative morbidity or mortality to the surgical procedure; pandemic effects during follow-up were addressed in sensitivity analyses. All baseline parameters, such as comorbidities, prior surgery or prior medications, were analyzed within two years before the index hospitalization. The baseline characteristics of the emulation compared to the original study are presented in Table B1.

#### Statistical methods to determine effect sizes of the emulation study

Table 1 presents all primary and secondary endpoints, which were checked for replicability with the treatment effect observed in the original studies^1^^2^. In accordance with the definitions of the original study, isolated removal of fixation material after LPF was not counted as a surgical complication, because this procedure may be elective and does not necessarily indicate treatment failure. In addition, surgical complications after discharge were only based on operative OPS codes. Therefore, non-operative manipulation or mobilization procedures were not part of the predefined endpoint definition and were not analysed separately. In general, all endpoints were classified and analysed as safety outcomes.

**Fig. 1:**
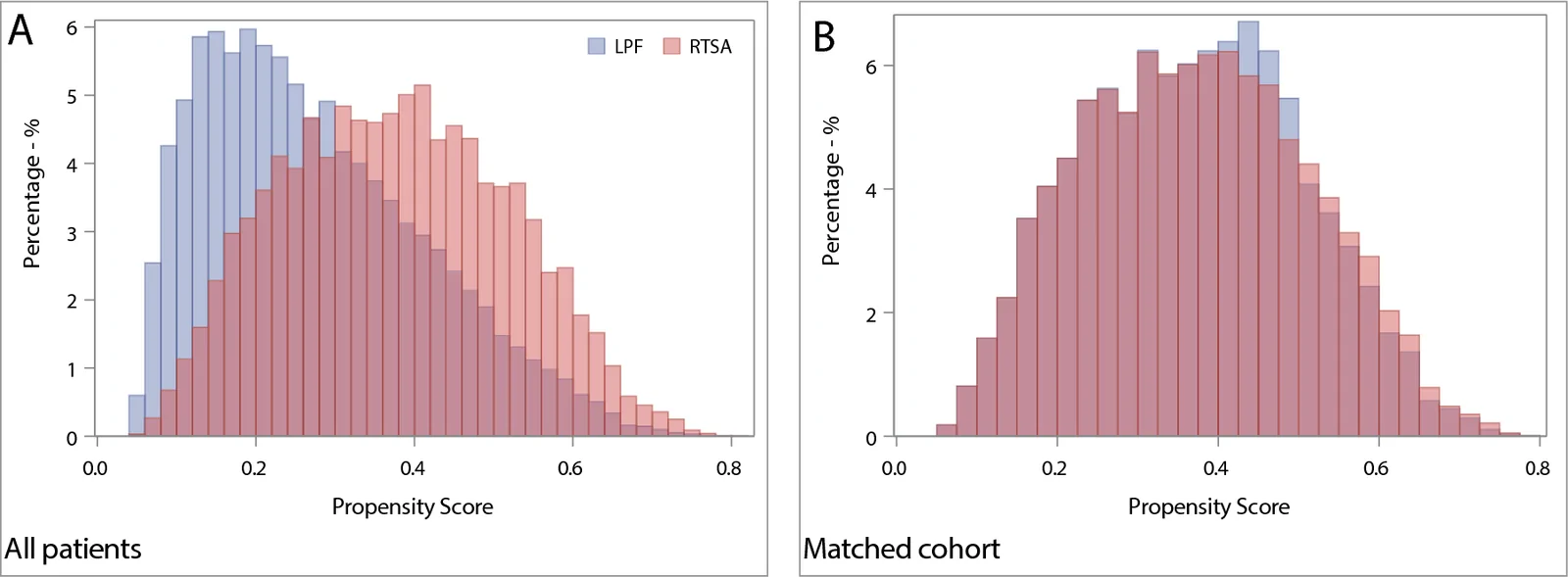
Overlap of propensity score for entire cohort before matching (**A**) and 1:1 matched cohort. LPF – locked plate fixation; RTSA – revere total shoulder arthroplasty.

To determine the effect sizes of the emulation, a 1:1 propensity score matching (PSM) including age, sex and entire patient’s risk profile was used. Balance between treatment groups were assessed using Z differences^49,50^, risk differences with 95% confidence interval (CI) and sum of squared Z differences (SSQZ$_{diff}$), where a threshold SSQZ$_{diff}$*<* “*number of included variables to PSM*” was considered to be sufficiently balanced (see Table 2). Moreover, the overlap of the propensity score is presented in Fig. 1.

**Table 1:** Summary of the analyzed primary and secondary endpoints.

Short-term^1^	Long-term^2^
Primary endpoints	
In-hospital surgical complication (SC; binary endpoints)	Surgical complications after discharge (defined as time from discharge to surgical complication)
In-hospital Major adverse events (MAE; binary endpoints)	MAE after discharge (defined as time from discharge to first MAE)
In-hospital thrombo-embolic events (TE; binary endpoint)	TE or death after discharge (defined as time from discharge to TE or death from any cause)
30-day mortality (binary endpoint)	Overall survival (defined as time from discharge to death from any cause)
Secondary endpoints	
Implant-assoc. complications (IAC; binary endpoint)	
Non-implant assoc. complications (NIAC; binary endpoint)	
	Minor outpatient complications (MOC; defined as time from discharge to minor outpatient complication)

Definitions can be found in^1,2^.

The effect sizes for the treatment effect of RTSA vs LPF were determined as odds ratios (ORs) for short-term endpoints and hazard ratios (HRs) for long-term endpoints. For all endpoints, multivariable logistic regression models (binary endpoints) and multivariable Cox regression models (time-to-event endpoints), including age, sex, year of surgery and patient’s individual risk profile, were used. In the case of surgical as well as minor outpatient complications after discharge, death was considered as a competing risk event and sub-distributional HRs using Fine&Gray Cox model were presented. The dependence of the matching pairs were considered using stratified analysis for all endpoints. Full regression results for all endpoints are presented in Table D3 – D13. In addition, all endpoints were presented descriptively, using proportions with 95% Clopper-Pearson confidence interval (binary variables), median and inter-quartile range (IQR, given by 25% quartile [Q1], and 75% quartile [Q3]; continuous variables) and event rates given by Aalen-Johansen estimates for the cumulative incidence functions (see Table C2). Statistical analyzes were performed using the SAS software V9.4 (SAS Institute Inc., Cary, NC, USA) and R version 4.2.0, R foundation, Vienna, Austria.

**Table 2:** Balance of the 1:1 propensity score matched cohort

Variable	LPF	RTSA	risk difference (95%CI)	Z difference
Sample size – n (%)	9,896 (50.0%)	9,896 (50.0%)	n.a.	n.a.
Mean age (± SD) – years	79.9 (± 7.4)	80.0 (± 6.9)	-0.1 (-0.3; 0.1)	1.048
Mean index year (± SD) – years	2018.2 (* ± 3.0 *)	2018.2 (* ± 2.9 *)	-0.03 (-0.1; 0.1)	0.895
Female sex – n (%)	8,651 (87.4%)	8,637 (87.3%)	0.1% (-0.8%; 1.1%)	-0.299
Comorbidities – n (%)				
Alcohol abuse	547 (5.5%)	549 (5.5%)	-0.0% (-0.7%; 0.6%)	0.062
Atrial fibrillation/ flutter	2,055 (20.8%)	2,135 (21.6%)	-0.8% (-2.0%; 0.3%)	1.392
Atherosclerosis	1,752 (17.7%)	1,748 (17.7%)	0.0% (-1.0%; 1.1%)	-0.075
Cancer	2,732 (27.6%)	2,769 (28.0%)	-0.4% (-1.6%; 0.9%)	0.587
Congestive heart failure	2,553 (25.8%)	2,669 (27.0%)	-1.2% (-2.4%; 0.1%)	1.871
Coronary heart disease	2,497 (25.2%)	2,557 (25.8%)	-0.6% (-1.8%; 0.6%)	0.978
Chronic kidney disease	2,852 (28.8%)	2,924 (29.6%)	-0.7% (-2.0%; 0.5%)	1.126
Chronic polyarthritis	676 (6.8%)	693 (7.0%)	-0.2% (-0.9%; 0.5%)	0.476
Dementia	1,098 (11.1%)	1,126 (11.4%)	-0.2% (-1.2%; 0.6%)	0.630
Diabetes Mellitus	3,255 (32.9%)	3,320 (33.6%)	-0.7% (-2.0%; 0.7%)	0.981
Frozen shoulder	433 (4.4%)	428 (4.3%)	0.1% (-0.1%; 0.6%)	-0.174
Hypertension	8,669 (87.6%)	8,654 (87.5%)	0.2% (-0.8%; 1.1%)	-0.323
Nicotine abuse	704 (7.1%)	695 (7.0%)	0.1% (-0.6%; 0.8%)	-0.250
Obesity	2,527 (25.5%)	2,520 (25.5%)	0.1% (-1.1%; 1.3%)	-0.114
Omarthrosis	380 (3.8%)	381 (3.9%)	0.0% (-0.5%; 0.5%)	0.037
Osteoporosis	4,053 (41.0%)	4,053 (41.0%)	0.0% (-1.4%; 1.4%)	0.000
Parkinson disease	369 (3.7%)	394 (4.0%)	-0.3% (-0.8%; 0.3%)	0.923
Stroke and/or CVD before PHF	2,651 (26.8%)	2,637 (26.7%)	0.1% (-1.1%; 1.4%)	-0.225
Rotator cuff rupture	495 (5.0%)	503 (5.1%)	-0.1% (-0.7%; 0.5%)	0.260
Any anticoagulant	3,196 (32.3%)	3,275 (33.1%)	-0.8% (-2.1%; 0.5%)	1.197
Analgesics	1,501 (15.2%)	1,584 (16.0%)	-0.8%(-1.9%; 0.2%)	1.627
Antibiotics	172 (1.7%)	190 (1.9%)	-0.2% (-0.6%; 0.2%)	0.955
Any anti-osteoporotic drugs	1,296 (13.1%)	1,319 (13.3%)	-0.2% (-1.1%; 0.7%)	0.483
			Caliper width	0.1
			Number of variables	26
			Mean of |*Z*|	0.650
			Sum of squared Z-differences	17.8

Balance between treatment groups were assessed using risk differences with 95% confidence interval, Z differences, mean of absolute Z differences and sum of squared Z-differences. CI – confidence interval; – CVD – cerebrovascular disease; LPF – locked plate fixation; RTSA – reverse total shoulder arthroplasty; SD – standard deviation.

#### Effect of COVID-19 pandemic

The original study was conducted prior to the SARS-CoV-2 pandemic. However, during the follow-up period of the study at hand, patients were influenced by the pandemic due to the infection itself on the one hand, but also influenced by lockdown periods and contact restrictions on the other hand. In addition, RTSA patients may had been affected more frequently due to an increasing proportion over study period. Thus, to address possible effects, two time-dependent variables were added to the Cox regression models. The first one reflects the beginning of the pandemic situation in Germany, and the second represent a diagnosed infection. As a sensitivity analysis, all regressions were repeated with COVID-19 infection were considered as a competing risk event and within a subset analysis including only patients with index surgery before 2020 ($N\,=\,10,879$, with end of follow-up defined as 31.12.2019; see supplement table E14 – E18).

### Statistical agreement metrics to evaluate the replicability

After determining all emulation effect sizes (EESs), the sceptical p-value was used to check the agreement between EESs and original effect sizes (OESs).

The two-trials rule (TTR) is a standardized request of the FDA in the context of pivotal trials. It is required that at least two independent studies confirm the efficacy of a new drug^51,52^. It is mostly implemented by two studies, which has to show significant results at a (one-sided) significance level of *α =0.025*^51^. The combined p-value of the two trials rule is then given by the maximum of the (one-sided) p-values of the two studies, i.e. $p_\text {TTR}\,=\, \max {\left( p_\text {O},\,p_\text {E}\right) }$. $p_\text {TTR}\,\le \, 0.025$ were interpreted as replication success. As an extension of the TTR, Held et al^46^ recently suggested a novel method that combines a reverse-Bayes approach with a prior-predictive assessment to detect conflicts^48,53^. The sceptical p-value $p_\text {scep}$ is influenced by the p-values of each study and the ratio of variances between OES and EES. Unlike treating the original and replication studies as interchangeable, this method imposes a penalty when the EES shrinks relative to the original one^47,48^. Moreover, $p_\text {scep}$ is an indirect measure of the degree of replication success^46^. It was shown that $p_\text {spec}$ can be re-calibrated in such a way that an exact Type-I error control is fulfilled at level $\alpha ^2$, if the effect is null in both studies^48^. The sceptical p-value is a one-sided p-value, to guarantee that replication is only successful, if both effects have the same sign. Further properties can be found in^46–48,51,53^. The controlled $p_\text {spec}$ was determined using R package *ReplicationSuccess* version 1.3.2, $p_\text {scep}\,\le \, 0.025$ were interpreted as replication success. Because the sceptical p-value assesses replication of a directional original effect, it was not applied when the original study showed no statistically significant treatment difference.

The following agreement metrics were additionally determined and compared to the conclusions from the sceptical p-value:

1. p-value from the TTR.
2. Regulatory agreement (RA): A binary agreement metric, which is 1, if the treatment effect estimates $\theta _O$ and $\theta _E$ (i.e. $\log (OR)\text { or }\log (HR)$) are pointing in the same direction and both studies report the same statistical significance on the same significance level, or 0 otherwise. In the case of an original null effect, regulatory agreement is fulfilled, when both studies report no significance at the same significance level^41,42^.
3. Estimated agreement (EA): EES is enclosed to the 95% CI of OES^41,42^.
4. Standardized difference agreement (SDA)^41,42^:
  1 $$\begin{aligned} \left| Z\right| \,\le \, 1.96,~\text {with}~ Z:=\,\frac{\hat{\theta }_{O}\,-\,\hat{\theta }_{E}}{\sqrt{\hat{\sigma }_{O}^2\,+\,\hat{\sigma }_{E}^2}}~, \end{aligned}$$
  where $\hat{\theta }_{O},\,\hat{\theta }_{E}$ are the treatment effect estimates and $\hat{\sigma }_{O}^2$, $\hat{\sigma }_{E}^2$ are the associated variance of the original trial and emulation study, respectively^41,42^.
5. Fixed effect meta-analysis to combine the original effects and replication study: For positive OES, replication is fulfilled, when the meta-analysis provides a significant combined effect^45^. In the case of an original null effect, replication is fulfilled, when the combined effect size using meta-analysis indicates also null^45^.

Moreover, Pearson’s correlation and intra-class correlation (ICC3 - two-way mixed) between EES and OES were calculated for a general overview.

**Fig. 2:**
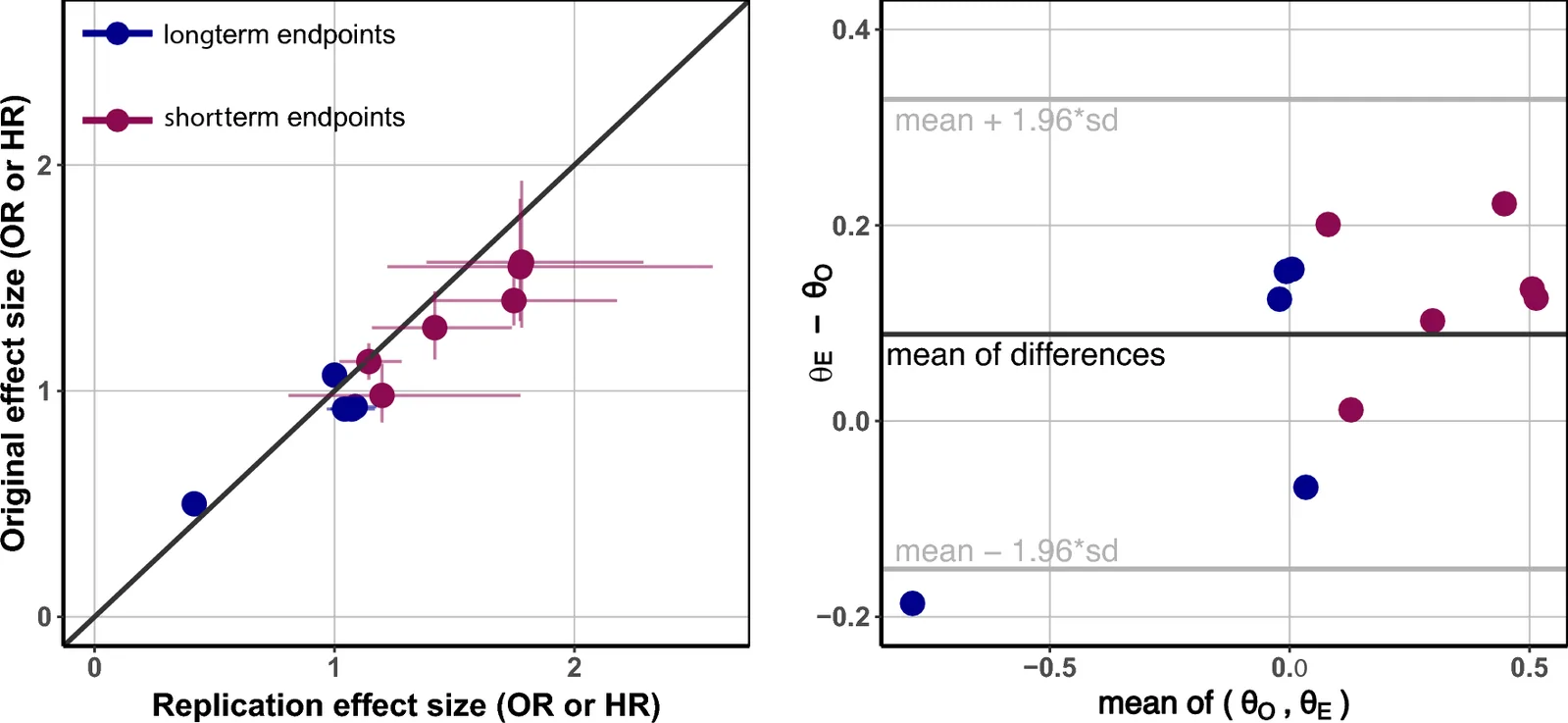
**Left:** Calibration plot. Original effect estimates (odds ratio –  OR or hazard ratio – HR) are plotted against the estimates of the emulation. **Right:** Bland Altman plot. The difference between the original and the emulation study model coefficients ($\theta _O \,-\,\theta _E$) are plotted against the averaged value for each pair.

## Results

Before PSM, N=32,952 patients were included to the emulation study. As presented in table B1, there are differences between the cohort of the original (AOK cohort) and emulation study (BARMER cohort). On the one hand, patients of the original study had more often a co-diagnosis of diabetes mellitus (AOK 40% vs BARMER 30%), of congestive heart failure (AOK 31% vs BARMER 23%) and of coronary heart disease (AOK 30% vs BARMER 24%) compared to the cohort of the emulation study. On the other hand, the emulation study had higher rates of patients with dementia (AOK 6% vs BARMER 10%) and higher rates of cancer (AOK 22% vs BARMER 27%). After 1:1 PSM, N=19,792 patients were included to the analysis set for further analysis.

Overall, 7 (64%) of the 11 evaluated endpoints were successfully replicated (s. table 3 and 4). The Pearson correlation coefficient of ($r_p$ = 0.97; 95% CI 0.88 –  0.99) and the intra-class correlation coefficient (single-fixed raters; ICC3 = 0.94; 95% CI 0.78 –  0.98) indicated a good, positive correlation between original and emulation study. The mean differences of effect estimates ($\theta _O \,-\,\theta _E$) was 0.089 (95% CI 0.006 –  0.171), with a slight increasing trend in the Bland-Altman plot (see Fig. 2). However, there were distinct differences for short- and long-term endpoints.

### Short-term endpoints

**Fig. 3:**
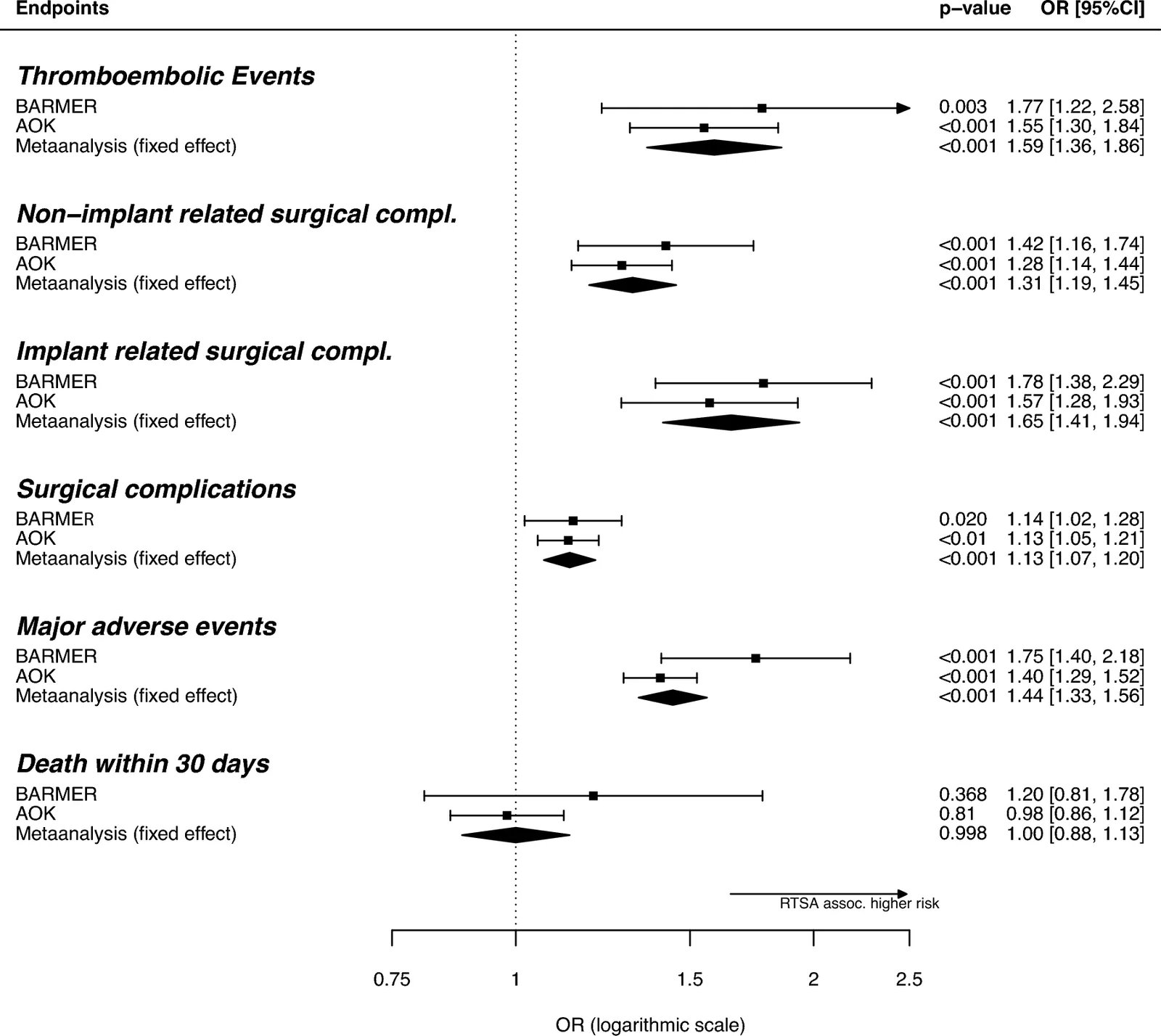
Short-term outcomes of original study (AOK^1^) and emulation study (BARMER). Combined effects determined using fixed-effect meta-analysis were included (diamond)

The effect estimates of the original study, the emulation study and the pooled effect from fixed-effect meta-analysis are presented in Fig. 3. For all analyzed endpoints, the pooled effect confirmed the results of the original study. RTSA was associated with higher risk for surgical complications (non-implant as well as implant-associated complications), higher risk for thrombo-embolic events and a higher risk for major adverse events during hospital stay (all *p<0.05*). In both studies, no differences in 30-day mortality after surgery between RTSA and LPF were observed. Moreover, almost all analyzed agreement metrics are fulfilled, except of 30-day mortality (see Table 3). Due to the original null effect for the 30-day mortality, the TTR resulted non-significant and the sceptical p-value cannot be determined. Nevertheless, the replication can also be considered successful, as various other criteria are met (see Table 3).

**Table 3:** Replication results for short-term outcomes

Agreement measure	30 day mortality	TE	MAE	SC	IAC	NIAC
Meta-analysis criterion fulfilled?	yes	yes	yes	yes	yes	yes
Regulatory agreement	yes	yes	yes	yes	yes	yes
Estimated agreement	no	yes	no	yes	yes	yes
SDA: *|Z|≤ 1.96*?	yes	yes	yes	yes	yes	yes
Standardized differences	0.948	0.643	1.85	0.168	0.758	0.853
$p_\text {TTR}$	0.610	0.001	<0.001	0.011	<0.001	<0.001
$p_\text {scep}$	n.a.$^\dagger$	<0.001	<0.001	0.006	<0.001	<0.001

$^\dagger$ not applicable for null effects. Standardized differences defined as $Z:=\,\frac{\hat{\theta }_{O}\,-\,\hat{\theta }_{E}}{\sqrt{\hat{\sigma }_{O}^2\,+\,\hat{\sigma }_{E}^2}}$, with $\theta =\log (OR)$. IAC – implant-associated complication; MAE – major adverse event; NIAC – non-implant associated complication; SC – surgical complication; TE – thrombo-embolic event.

### long-term endpoints

**Table 4:** Replication results for long-term outcomes

Agreement measure	OS	TE	MAE	SC	MOC
Meta-analysis criterion fulfilled?	yes	yes	yes	yes	yes
Regulatory agreement	no	no	no	yes	no
Estimated agreement	no	no	no	no	no
SDA: *|Z|≤ 1.96*?	no	no	no	no	no
Standardized differences	3.177	3.592	3.024	-2.744	-2.037
$p_\text {TTR}$	0.942	0.980	0.864	<0.001	0.998
$p_\text {scep}$	0.961	0.990	0.888	<0.001	0.512

Standardized differences defined as $Z:=\,\frac{\hat{\theta }_{O}\,-\,\hat{\theta }_{E}}{\sqrt{\hat{\sigma }_{O}^2\,+\,\hat{\sigma }_{E}^2}}$, with $\theta =\log (HR)$. MAE – major adverse event; MOC – minor outpatient complication; OS – overall survival; SC – surgical complication; TE – thrombo-embolic event.

**Fig. 4:**
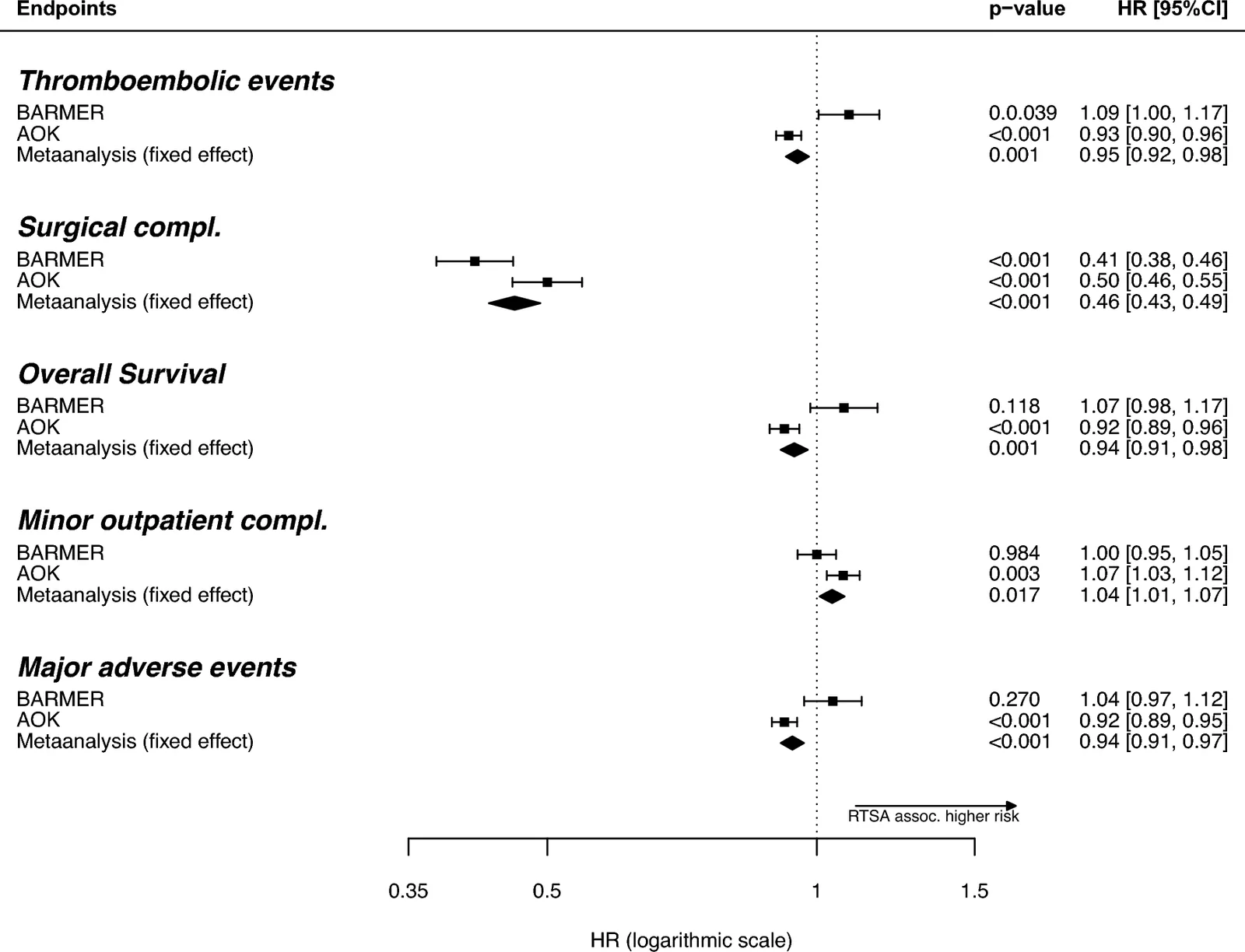
Long-term outcomes of original study (AOK,^2^) and emulation study (BARMER). Combined effects determined using fixed-effect meta-analysis were included (diamond)

In total, N=15.518 were included for the analysis of the follow-up outcome with exclusion criteria presented in Fig. A1. The effect estimates of the original study, the emulation study and the pooled effect from fixed-effect meta-analysis are presented in Fig. 4. For surgical complications after discharge, the original treatment effect was successfully replicated, because $p_\text {scep}<0.001$, $p_\text {TTR}<0.001$ and the fixed-effect meta-analysis confirmed the original treatment effect (see Table 4). Since the treatment effect on surgical complications after discharge was even more pronounced in the emulation study (RTSA vs LPF: HR 0.41, 95%CI 0.38 – 0.46, p<0.001), the 95%CIs of the OES and EES did not overlap and EA and SDA were thus not fulfilled. One year after initial LPF, 3.4% (95%CI 3.2 – 3.6%) of patients had undergone conversion to RTSA (see Table C2). For all other evaluated endpoints during follow-up, the replication was not successful, even if the pooled effects from meta-analysis were in accordance with the results of the original findings (see Fig. 4 and Table 4).

## Discussion

For the implemented emulation study, all studied short-term outcomes and the treatment effect on surgical complication in the long-term after surgical treatment of PHF using LPF or RTSA were successfully replicated. The sceptical p-value was a well interpretable metric to judge the agreement of different studies also in the context of RWE. While all endpoints are treated as safety outcomes in this analysis, surgical complications occurring during follow-up merit a dual interpretation. We note that all observed surgical complications were defined as secondary surgeries or procedural interventions. However, surgical complications after discharge may also indicate reduced treatment durability because they represent the need for additional operative or procedural treatment of the shoulder. Because claims data do not capture patient-reported function or symptom severity, we report these events as safety outcomes, while noting that their occurrence can also serve as an administrative signal of subsequent treatment failure.

The different agreement metrics have different advantages and disadvantages, which can lead to different judgments. For all analyzed endpoints, the TTR and the sceptical p-value came to the same conclusion. However, purely assessing the significance of the original and replication studies (TTR) is often used as a criterion, but it has the significant disadvantage of not considering the effect sizes of both, potentially leading to incorrect conclusions^48^. Additionally, the TTR has other major limitations, such as low project power or the inability to achieve successful replicability if the p-value of the original study is even slightly above the 5% mark (or 2.5% for one-sided hypotheses)^48^. However, replicability of results that show a trend towards significance (i.e., barely missing the mark) is common practice, which can be also addressed using the sceptical p-value^48^.

In the case of positive treatment effects, both, regulatory agreement as well as the TTR, are equivalent to each other. But, for null effects, the TTR is per definition not fulfilled, even though the second (emulation) study concluded also a null effect, which leads to discrepancies between the binary metric and the combined p-value. Furthermore, the sceptical p-value cannot be used for an original null effect and only binary agreement metrics (i.e. RA, EA or SDA) or a meta-analytic approach are available. Micheloud et al.^54^ presented a formalism for equivalence studies. However, when the original study was designed for superiority and a non-significant effect was observed (null-effect), it should not be confused with an equivalence test, as “*the absence of evidence is not evidence for absence*”^55^. However, in the case of an original null effect, it would be more appropriate to initiate a new study with the aim of proving the equivalence between treatments (with an equivalence test as the primary analysis) instead of starting a (further) replication study with the “wrong design”.

Meta-analytic approaches, could be useful to determined pooled effect estimates over different studies or different cohorts (i.e. different insurance companies). However, to evaluate the replication of a known effect, this approach has some distinct disadvantages. On the one hand, it is assumed that the studies are independent and exchangeable, which is not fulfilled in the case of a setting of original and emulation study^46,51^. On the other hand, when the sample size of the original study is much larger than the one of the emulation study, it is possible that the meta-analytic approach confirm the original effect, even if the emulation study concluded qualitatively different effect estimates. This was the case for the studied long-term endpoints OS, MAE, TE and MOC where the 95%CI of the original effect estimates where smaller (for MOC greater) than one (all p<0.001), but the emulation failed to show any differences for RTSA compared to LPF.

Especially in the case of emulating RCTs using RWD, the regulatory, estimated and standardized difference agreement were common and often used^41,42,56–61^. However, the sceptical p-value has some advantages over the binary metrics, which were in detail described in^62^. In practical terms, the sceptical p-value provides a single directional and graded assessment of replication that accounts for uncertainty in both studies and penalizes conflict or marked shrinkage, while not declaring failure when the emulation confirms the original effect even more strongly. In the presented study, EA and SDA were not fulfilled for surgical complications during long-term, as the 95% CIs of the original and emulated treatment effect did not overlap. Based on SDA and EA, one might conclude that the replication failed, even though the original effect was confirmed even more clearly. In contrast, the sceptical p-value leads to the correct conclusion.

As observed by Koeppe et al.^1^, RTSA was associated with higher risk for different complications during hospital stay, but no differences were found for 30-day mortality. Moreover, the findings for surgical complications after discharge observed by Stolberg-Stolberg et al.^2^ could also be replicated, i.e. RTSA compared to LPF was associated with a lower risk for surgical complications after discharge. Heyard et al.^43^ used meta-analytic methods to identify reasons for RWE results not being consistent with original RCT effects. They concluded that the most heterogeneity between RCT and related RWE emulation are caused by delayed effect of treatment, discontinuation of treatment during run-in period and treatment started in hospital. Wang et al.^38^ concluded that failure of direct replication was mostly explained by incomplete or intransparent reporting and updated data. However, none of the possible explanations reported in Heyard et al.^43^ or Wang et al.^38^ were present in the original studies, suggesting that a different source for the discrepancies must be assumed. As a potential explanation, the failure of replication success for the long-term endpoints overall survival, major adverse events and thrombo-embolic events could be caused by missing causal relationship between the treatment of PHF and events in the much later course of the follow-up. This is also underlined by the fact that, even years later, the occurrence of secondary shoulder surgery is very likely to be directly related to the past fracture and treatment and that the results for this endpoint (SC) of the original study were successfully replicated and thus confirmed. Moreover, in the emulation, RTSA was even more clearly associated with less SCs than in the original study. However, even if there are statistically noticeable differences for OS, MAE and TE between original and emulation study, the observed effect is only of limited clinical relevance and, therefore, of secondary interest in the interpretation of content. In contrast, during hospital stay, all endpoints were successfully replicated, where the causal relationship between surgery and all endpoints seemed not unlikely. This may also indicate that replication is only successful, if a direct causal relationship between the treatment effect (to be tested) and the endpoint can be assumed. For the discrepant long-term systemic endpoints, residual treatment selection due to unmeasured clinical factors and remaining differences between the AOK and BARMER populations may have contributed. Competing mortality was partly addressed by design: death was the endpoint for overall survival, part of the major adverse and thrombo-embolic events, and was treated as a competing risk for surgical complications and minor outpatient complications. Nevertheless, differential mortality and frailty may still affect interpretation of long-term non-fatal outcomes.

The manuscript addresses a common and challenging treatment decision in older patients with proximal humeral fractures. It confirms that RTSA is associated with higher short-term complication rates but fewer long-term re-operations compared to LPF as well as the need for individualized treatment algorithms, highlighting an important clinical trade-off. 

### Implications for practice

 The replicated short-term in-hospital safety signals (higher perioperative complication rates with RTSA) and the replicated finding of lower subsequent shoulder surgery after RTSA together suggest a clinically relevant trade-off that clinicians should discuss with older patients facing operative treatment for proximal humeral fractures. Because our analyses are based on claims data and remain observational despite matching and adjustment, these findings should inform shared decision-making and perioperative planning, but not be interpreted as definitive evidence to mandate a particular surgical strategy. Future integration of claims data with clinical registries and patient-reported outcome measures would help to further guide treatment choice. 

### Methodological implications for RWE replication

 In general, replication of previous observed treatment effects is essential to increase the evidence of findings, especially in the context of real-world data studies. The sceptical p-value, originally developed for pivotal trials, also provides the opportunity for a comprehensive assessment of replication in the context of real-world evidence, which was demonstrated on the presented data and by Koeppe et al.^62^. Moreover, it is essential once again that the information on the design, definition of the cohort (including inclusion and exclusion criteria) and variables, as well as the statistical analysis, are transparent and fully available. The presented study illustrated how an evaluation of the agreement can further improve the evidence and validity of results from real-world data. 

### Strengths and limitations

 There are some limitations that have to be considered and are caused by the retrospective character of the study. First, as in the original study, the data are insurance data primarily collected for financial purposes. The database does not provide information about the underlying reason for the respective treatment with RTSA or LPF. Moreover, functional outcomes (pain, range of motion, health-related quality of life) are not available in claims data and are therefore not assessed. Residual confounding from unmeasured clinical factors (e.g. fracture morphology/displacement, bone quality, baseline function/frailty, and provider/hospital experience/volume) is possible in claims-data analyses and may particularly affect interpretation, especially of longer-term systemic outcomes.. The sceptical p-value is not applicable to replicate an observed null effect.

## Conclusions

All treatment effects of the original study for short-term safety endpoints and for surgical complications during follow-up were successfully replicated using an independent data base. These results suggest a trade-off between short-term safety and longer-term need for reoperation that should be explicitly discussed with patients. The sceptical p-value was a very useful tool to evaluate the agreement between original and emulation study.

## Supplementary Information

Supplementary Information.

## Data Availability

The datasets generated and/or analysed during the current study are not publicly available due to German data protection laws (‘Bundesdatenschutzgesetz’, BDSG). They are stored on a server of the BARMER Institute for Health System Research, to facilitate replication of the results. In general, access to data of statutory health insurance funds for research purposes is possible only under the conditions defined in German Social Law (SGB V §287) and further inquiries can be directed to the corresponding author on reasonable request. All aggregated data required to evaluate the replication claims, including cohort counts, balance diagnostics, effect estimates, confidence intervals, p-values, and agreement metrics, are provided in the manuscript and Supplementary Material.

## Abbreviations

CI: Confidence interval
CONSORT: Consolidated standards of reporting trials
COVID-19: Corona virus disease 2019
EES: Emulation effect estimate
HR: Hazard ratio
IAC: Implant-associated complications
ICD-10 GM: International statistical classification of diseases, German modification
IQR: Inter quartile range
LPF: Locked plate fixation
MAE: Major adverse event
MOC: Minor outpatient complications
NIAC: Non-implant associated complications
OES: Original effect size
OPS: *Operationen- und Prozeduren-Schlüssel* (German procedure classification)
OR: Odds ratio
PHF: Proximal humeral fracture
PI: Prediction interval
RCT: Randomized controlled trial
RWD: Real-world data
RWE: Real-world evidence
RTSA: Reverse total shoulder arthroplasty
SC: Surgical complications
TE: Thromboembolic event
