# Supplementary material for: The replicability of real-world evidence for the surgical treatment of proximal humeral fractures in older patients evaluated using sceptical p-value
Source: Sci Rep. 2026 Sep 8;16:28068. doi: 10.1038/s41598-026-70157-6 (PMC13554206; doi:10.1038/s41598-026-70157-6)
Supplement: Supplementary file 1 [file 41598_2026_70157_MOESM1_ESM.pdf]

Supplementary information

Appendix A CONSORT flow-chart of the emulation study

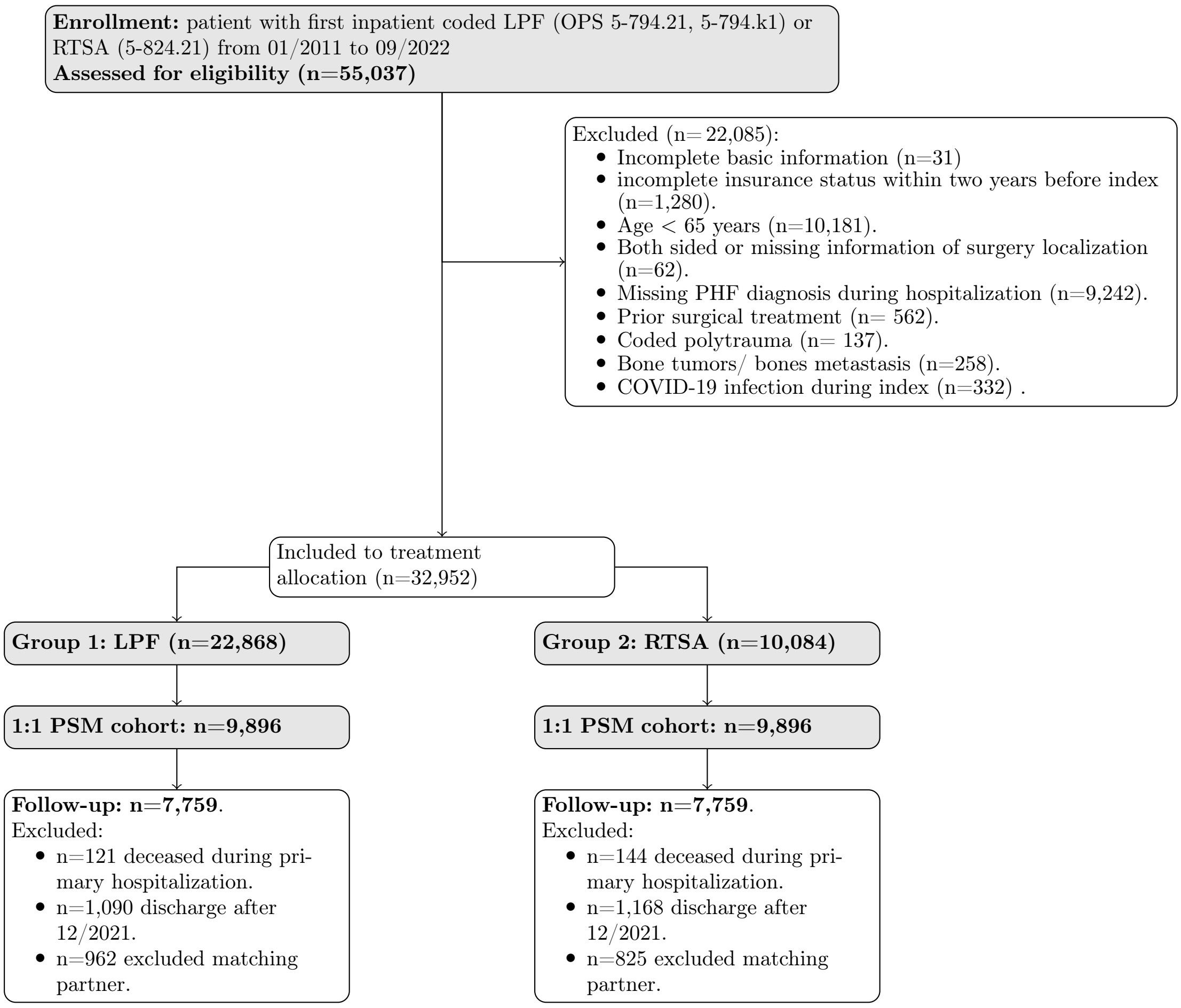

Fig. A1: CONSORT flow chart of the emulation study. LPF – locked plate fixation; PHF – proximal humeral fracture; PSM – propensity score matching; RTSA – revere total shoulder arthroplasty.

Appendix B Patient characteristics replication study before matching

.

Table B1: Patient characteristics of the replication study for PHF compared to the original study cohort presented in [1]

	**Emulation study cohort**		**Original study cohort: [1]**	
	LPF	RTSA	LPF	RTSA
**Frequency – n (%)**	**22,868 (69%)**	**10,084 (31%)**	**41,216 (74.8%)**	**13,854 (25.2%)**
Median age – years (IQR)	77 (12)	80 (10)	78 (11)	81 (9)
Age $\geq$ 80 years – % (n)	37% (8,556)	54% (5,475)	43% (17,857)	56% (7,785)
Female sex – % (n)	86% (19,621)	87% (8,809)	84% (34,476)	87% (12,012)
**Comorbidities – % (n):**				
Osteoporosis	36% (8,163)	42% (4,185)	35% (14,466)	41% (5,639)
Cancer	27% (6,107)	28% (2,835)	22% (8,996)	23% (3,192)
Diabetes Mellitus	29% (6,602)	34% (3,406)	39% (16,191)	43% (5,965)
Dementia	10% (2,262)	11% (1,157)	5% (2,199)	6% (835)
Chronic polyarthritis	6% (1,452)	7% (708)	6% (2,505)	6%(873)
Omarthrosis	3% (654)	4% (394)	2% (873)	3% (392)
Obesity	22% (4,978)	26% (2,590)	27% (11,073)	32% (4,481)
Nicotine abuse	7% (1,632)	7% (700)	6% (2,500)	5% (667)
Parkinson disease	4% (805)	4% (401)	4% (1,668)	4% (568)
Rotator cuff rupture	4% (816)	5% (519)	6% (2,621)	10% (1,369)
Alcohol abuse	5% (1,216)	6% (560)	6% (2,294)	5% (723)
Stroke and/or CVD before PHF	25% (5,631)	27% (2,699)	25% (10,262)	27% (3,692)
Frozen shoulder	4% (990)	4% (436)	4% (1,838)	5% (624)
Atrial fibrillation/ flutter	16% (3,758)	22% (2,197)	18% (7,512)	23% (3,248)
Congestive heart failure	21% (4,832)	27% (2,763)	29% (12,070)	35% (4,885)
Coronary heart disease	23% (5,328)	26% (2,626)	30% (12,471)	33% (4,615)
Hypertension	82% (18,742)	88% (8,834)	87% (35,830)	91% (12,644)
Atherosclerosis	16% (3,579)	18% (1,780)	17% (6,815)	19% (2,624)
Chronic kidney disease	22% (5,095)	30% (3,026)	26% (10,857)	35% (4,824)
**Prev. drugs – % (n):**				
Any anticoagulant	28% (6,309)	33% (3,368)	27% (11,299)	33% (4,519)
Analgesics[1]	14% (3,205)	16% (1,615)	8% (3,340)	8% (1,109)
Antibiotics[1]	2% (490)	2% (192)	1% (517)	1% (135)
Vitamin D or Calcium	7% (1,537)	8% (830)	4% (1,668)	6% (763)
Bisphosphonates	7% (1,585)	7% (715)	7% (2,837)	7% (1,026)

Definitions of all parameters can be found in [1, 2].

[1]Only within 14 days before admission.

CVD – cerebrovascular disease; IQR – inter-quartile range; LPF – locked plate fixation; PHF – proximal humeral fracture; RTSA – reverse total shoulder arthroplasty.

Appendix C Univariate outcome of the replication study

Table C2: Univariate outcome of the replication study for PHF compared to the original study cohort presented in [1, 2, 63].

	**Emulation study cohort**		**Original study cohort**	
	LPF	RTSA	LPF	RTSA
	**Inhospital outcomes**			
Mean LOS – day (± SD)	11.5 (±8.2)	15.2 (±8.8)	14.6 (±11.4)	20.0 (±13.5)
Mean charges – EUR (± SD)	6895.0 (±5139.0)	11, 200.8 (±4846.0)	7030.1 (±5532.0)	11, 165.7 (±5884.4)
MAEs – % (95%CI)	3.2% (3.0%; 3.4%)	6.2% (5.7%; 6.7%)	4%	7%
TEs – % (95%CI)	0.6% (0.5%; 0.7%)	1.0% (0.8%; 1.2%)	1%	2%
SC – %(95%CI)	6.5% (6.2%; 6.8%)	7.4% (6.9%; 7.9%)	8%	9%
Implant-assoc. compl. – %(95%CI)	1.0% (0.9%; 1.2%)	2.3% (2.0%; 2.6%)	1%	1%
Non-implant assoc. compl. – %(95%CI)	2.0% (1.9%; 2.2%)	2.7% (2.4%; 3.0%)	3%	3%
deep infection – %(95%CI)	0.6% (0.5%; 0.7%)	0.7% (0.5%; 0.9%)	1%	1%
Death (any cause) – % (95%CI)	0.9% (0.8%; 1.1%)	1.5% (1.3%; 1.7%)	2%	2%
30-day mortality – % (95%CI)	1.5% (1.3%; 1.6%)	2.2% (2.0%; 2.5%)	2%	3%
	**Long-term outcomes**			
**OS after discharge**				
1 year	6.4% (6.1%; 6.7%)	8.7% (8.1%; 9.3%)	8.4% (8.1%; 8.6%)	9.1% (8.7%; 9.7%)
5 years	28.0% (27.3%; 28.7%)	36.8% (35.5%; 38.0%)	34.5% (34.0%; 35.1%)	39.4% (38.2%; 40.7%)
**MAEs after discharge**				
1 year	10.4% (10.0%; 10.8%)	13.6% (12.9%; 14.4%)	12.4% (12.1%; 12.8%)	14.2% (13.6%; 14.8%)
5 years	37.6% (36.8%; 38.3%)	47.5% (46.2%; 48.8%)	44.7% (41.4%; 45.3%)	50.0% (48.7%; 51.2%)
**TEs after discharge**				
1 year	8.7% (8.3%; 9.1%)	11.6% (10.9%; 12.3%)	11.0% (10.7%; 11.3%)	12.2% (11.6%; 12.8%)
5 years	33.5% (32.8%; 34.2%)	42.5% (41.2%; 43.8%)	40.9% (40.3%; 41.5%)	45.9% (44.7%; 47.2%)
**SC after discharge**				
1 year	11.6% (11.2%; 12.1%)	4.0% (3.6%; 4.4%)	9.5% (9.3%; 9.8%)	4.0% (3.6%; 4.3%)
5 years	15.8% (15.3%; 16.3%)	8.0% (7.3%; 8.6%)	12.0% (11.7%; 12.3%)	6.2% (5.7%; 6.7%)
**MOC after discharge**				
1 year	24.1% (23.5%; 24.7%)	23.3% (22.4%; 24.2%)	20.8% (20.4%; 21.2%)	21.9% (21.2%; 22.6%)
5 years	34.2% (33.5%; 34.9%)	33.6% (32.6%; 34.7%)	30.6% (30.1%; 31.1%)	31.7% (30.8%; 32.6%)
**Conversion to RTSA**				
1 years	3.4% (3.2%; 3.6%)	–	2.7% (2.6%; 2.9%)	–
5 years	4.7% (4.4%; 4.9%)	–	3.7% (3.5%; 3.9%)	–

Definitions of all parameters can be found in [1, 2]. For binary endpoints (short-term endpoints) of the replication cohort, ratios with 95% Clopper-Pearson confidence interval (CI) are presented. For time-to-event endpoints, event rates were given by the cumulative incidence function determined using Aalen-Johansen estimate with 95%-CI.

LOS – length of hospital stay, LPF – locked plate fixation, MAE – major adverse event, MOC – minor outpatient complications, TE– thrombo-embolic events, SC – surgical complications, SD– standard deviation, RTSA – reverse total shoulder arthroplasty.

Appendix D Full regression results for all presented models

Table D3: Results from logistic regression model – major adverse events.

Variable	OR	95% CI	p-value (two-sided)
Treatment group (RTSA vs LPF)	1.75	1.40 – 2.19	<0.001
Age at index surgery (years)	0.95	0.67 – 1.35	0.759
Male sex	2.08	0.70 – 6.18	0.186
Year of index surgery (years)	0.72	0.23 – 2.30	0.584
Any anticoagulant	1.04	0.78 – 1.38	0.790
Any anti-osteoporotic drug	0.98	0.58 – 1.66	0.934
Cancer	1.15	0.85 – 1.56	0.356
Diabetes mellitus	0.78	0.27 – 2.21	0.637
Dementia	1.92	1.03 – 3.59	0.040
Chronic polyarthritis	1.22	0.64 – 2.30	0.550
Obesity	0.45	0.08 – 2.73	0.388
Nicotine abuse	1.51	0.91 – 2.50	0.113
Parkinson disease	1.02	0.45 – 2.32	0.964
Rotator cuff rupture	0.31	0.02 – 5.38	0.417
Alcohol abuse	0.91	0.13 – 6.60	0.927
Atrial fibrillation/ flutter	1.39	0.78 – 2.47	0.260
Hypertension	1.02	0.32 – 3.26	0.970
Congestive heart failure	2.11	1.12 – 3.97	0.021
Coronary heart disease	1.61	1.08 – 2.39	0.020
Atherosclerosis	1.08	0.76 – 1.53	0.682
Chronic kidney disease	1.86	1.23 – 2.83	0.004
Stroke and/or CVD before PHF	1.13	0.71 – 1.80	0.608
Omarthrosis	0.31	0.08 – 1.29	0.108
Frozen shoulder	0.74	0.33 – 1.62	0.446
Analgesics*	0.62	0.45 – 0.87	0.005
Antibiotics*	1.68	0.55 – 5.10	0.363

*only within 14 days before admission. CVD – cerebrovascular disease, LPF – locked plate fixation, OR – odds ratio, PHF – proximal humeral fracture, RTSA – reverse total shoulder arthroplasty.

Table D4: Results from logistic regression model – thrombo-embolic events.

Variable	OR	95% CI	p-value (two-sided)
Treatment group (RTSA vs LPF)	1.77	1.22 – 2.58	0.003
Male sex	0.97	0.42 – 2.22	0.939
Age at index surgery (years)	0.91	0.85 – 0.97	0.005
Year of index surgery (years)	0.71	0.66 – 0.77	<0.001
Any anticoagulant	0.88	0.46 – 1.68	0.697
Any anti-osteoporotic drug	1.16	0.49 – 2.71	0.736
Cancer	1.29	0.73 – 2.29	0.386
Diabetes mellitus	0.71	0.40 – 1.27	0.245
Dementia	2.56	1.16 – 5.66	0.020
Chronic polyarthritis	1.90	0.59 – 6.14	0.284
Obesity	0.50	0.24 – 1.00	0.051
Nicotine abuse	0.88	0.31 – 2.45	0.800
Parkinson disease	0.82	0.14 – 4.91	0.824
Rotator cuff rupture	0.27	0.08 – 0.86	0.027
Alcohol abuse	0.17	0.04 – 0.86	0.032
Atrial fibrillation/ flutter	1.52	0.72 – 3.20	0.271
Hypertension	0.50	0.16 – 1.51	0.217
Congestive heart failure	1.21	0.64 – 2.31	0.563
Coronary heart disease	1.97	1.01 – 3.84	0.046
Atherosclerosis	1.18	0.55 – 2.50	0.676
Chronic kidney disease	0.70	0.37 – 1.32	0.268
Stroke and/or CVD before PHF	1.59	0.90 – 2.81	0.107
Omarthrosis	0.35	0.08 – 1.47	0.153
Frozen shoulder	0.74	0.20 – 2.67	0.644
Analgesics*	1.17	0.56 – 2.44	0.684
Antibiotics*	0.57	0.10 – 3.46	0.544

*only within 14 days before admission. CVD – cerebrovascular disease, LPF – locked plate fixation, OR – odds ratio, PHF – proximal humeral fracture, RTSA – reverse total shoulder arthroplasty.

Table D5: Results from logistic regression model – surgical complications.

Variable	OR	95% CI	p-value (two-sided)
Treatment group (RTSA vs LPF)	1.14	1.02 – 1.28	0.021
Male sex	2.67	2.07 – 3.45	<0.001
Age at index surgery (years)	0.91	0.89 – 0.92	<0.001
Year of index surgery (years)	0.71	0.70 – 0.73	<0.001
Any anticoagulant	1.27	1.03 – 1.57	0.023
Any anti-osteoporotic drug	1.23	0.96 – 1.57	0.104
Cancer	1.18	0.98 – 1.42	0.083
Diabetes mellitus	0.74	0.62 – 0.89	0.001
Dementia	1.56	1.21 – 2.01	<0.001
Chronic polyarthritis	0.98	0.71 – 1.35	0.909
Obesity	0.61	0.50 – 0.76	<0.001
Nicotine abuse	0.98	0.71 – 1.34	0.889
Parkinson disease	1.17	0.80 – 1.72	0.420
Rotator cuff rupture	0.70	0.51 – 0.96	0.025
Alcohol abuse	0.78	0.54 – 1.11	0.171
Atrial fibrillation/ flutter	0.91	0.72 – 1.14	0.393
Hypertension	0.73	0.55 – 0.96	0.025
Congestive heart failure	0.93	0.76 – 1.15	0.513
Coronary heart disease	0.91	0.75 – 1.12	0.387
Atherosclerosis	1.03	0.84 – 1.28	0.760
Chronic kidney disease	1.12	0.92 – 1.35	0.259
Stroke and/or CVD before PHF	1.14	0.94 – 1.37	0.194
Omarthrosis	0.88	0.59 – 1.31	0.517
Frozen shoulder	0.93	0.63 – 1.38	0.719
Analgesics*	0.95	0.76 – 1.18	0.615
Antibiotics*	1.21	0.63 – 2.29	0.568

*only within 14 days before admission. CVD – cerebrovascular disease, LPF – locked plate fixation, OR – odds ratio, PHF – proximal humeral fracture, RTSA – reverse total shoulder arthroplasty.

Table D6: Results from logistic regression model – implant-associated complications.

Variable	OR	95% CI	p-value (two-sided)
Treatment group (RTSA vs LPF)	1.78	1.38 – 2.29	<0.001
Male sex	3.97	2.26 – 6.96	<0.001
Age at index surgery (years)	0.85	0.82 – 0.89	<0.001
Year of index surgery (years)	0.71	0.69 – 0.74	<0.001
Any anticoagulant	0.97	0.61 – 1.54	0.895
Any anti-osteoporotic drug	1.17	0.66 – 2.10	0.590
Cancer	1.26	0.83 – 1.92	0.286
Diabetes mellitus	0.57	0.38 – 0.85	0.005
Dementia	2.13	1.24 – 3.67	0.007
Chronic polyarthritis	1.20	0.60 – 2.38	0.612
Obesity	0.66	0.42 – 1.04	0.075
Nicotine abuse	0.88	0.46 – 1.66	0.684
Parkinson disease	1.60	0.69 – 3.72	0.279
Rotator cuff rupture	1.02	0.51 – 2.07	0.947
Alcohol abuse	0.75	0.33 – 1.71	0.489
Atrial fibrillation/ flutter	0.86	0.52 – 1.45	0.577
Hypertension	0.41	0.21 – 0.81	0.010
Congestive heart failure	1.01	0.65 – 1.58	0.958
Coronary heart disease	0.88	0.57 – 1.34	0.545
Atherosclerosis	1.32	0.84 – 2.08	0.230
Chronic kidney disease	1.42	0.94 – 2.14	0.098
Stroke and/or CVD before PHF	1.30	0.86 – 1.97	0.220
Omarthrosis	0.93	0.43 – 2.02	0.863
Frozen shoulder	0.45	0.20 – 1.00	0.049
Analgesics*	1.27	0.78 – 2.06	0.341
Antibiotics*	1.13	0.31 – 4.09	0.854

*only within 14 days before admission. CVD – cerebrovascular disease, LPF – locked plate fixation, OR – odds ratio, PHF – proximal humeral fracture, RTSA – reverse total shoulder arthroplasty.

Table D7: Results from logistic regression model – non-implant associated complications.

Variable	OR	95% CI	p-value (two-sided)
Treatment group (RTSA vs LPF)	1.42	1.16 – 1.74	<0.001
Male sex	2.53	1.57 – 4.07	<0.001
Age at index surgery (years)	0.94	0.91 – 0.97	<0.001
Year of index surgery (years)	0.71	0.69 – 0.73	<0.001
Any anticoagulant	1.37	0.95 – 1.97	0.093
Any anti-osteoporotic drug	1.45	0.90 – 2.35	0.128
Cancer	1.27	0.90 – 1.79	0.171
Diabetes mellitus	0.66	0.48 – 0.92	0.015
Dementia	1.69	1.07 – 2.65	0.023
Chronic polyarthritis	0.95	0.51 – 1.78	0.869
Obesity	0.39	0.26 – 0.59	<0.001
Nicotine abuse	1.85	0.99 – 3.45	0.053
Parkinson disease	0.88	0.42 – 1.82	0.723
Rotator cuff rupture	0.58	0.31 – 1.08	0.086
Alcohol abuse	0.91	0.49 – 1.68	0.752
Atrial fibrillation/ flutter	1.32	0.89 – 1.96	0.171
Hypertension	0.80	0.48 – 1.35	0.406
Congestive heart failure	0.77	0.53 – 1.12	0.169
Coronary heart disease	0.94	0.65 – 1.36	0.734
Atherosclerosis	1.30	0.89 – 1.91	0.176
Chronic kidney disease	1.11	0.79 – 1.57	0.551
Stroke and/or CVD before PHF	1.18	0.83 – 1.67	0.369
Omarthrosis	1.52	0.73 – 3.19	0.265
Frozen shoulder	2.15	0.98 – 4.70	0.055
Analgesics*	0.98	0.65 – 1.47	0.912
Antibiotics*	1.96	0.44 – 8.73	0.379

*only within 14 days before admission. CVD – cerebrovascular disease, LPF – locked plate fixation, OR – odds ratio, PHF – proximal humeral fracture, RTSA – reverse total shoulder arthroplasty.

Table D8: Results from logistic regression model – 30-day mortality.

Variable	OR	95% CI	p-value (two-sided)
Treatment group (RTSA vs LPF)	1.20	0.81 – 1.78	0.368
Male sex	1.18	0.19 – 7.34	0.859
Age at index surgery (years)	1.46	0.79 – 2.69	0.224
Year of index surgery (years)	2.35	0.32 – 17.24	0.400
Any anticoagulant	1.24	0.72 – 2.13	0.436
Any anti-osteoporotic drug	0.94	0.37 – 2.37	0.898
Cancer	0.91	0.54 – 1.55	0.735
Diabetes mellitus	3.02	0.52 – 17.61	0.220
Dementia	1.09	0.37 – 3.18	0.880
Chronic polyarthritis	1.42	0.42 – 4.78	0.568
Obesity	3.10	0.14 – 68.80	0.474
Nicotine abuse	2.51	1.02 – 6.17	0.046
Parkinson disease	1.77	0.43 – 7.33	0.431
Rotator cuff rupture	3.93	0.03 – 561.0	0.589
Alcohol abuse	5.18	0.17 – 160.4	0.348
Atrial fibrillation/ flutter	3.29	1.28 – 8.47	0.014
Hypertension	1.66	0.23 – 11.79	0.613
Congestive heart failure	4.33	1.41 – 13.25	0.010
Coronary heart disease	1.09	0.54 – 2.23	0.808
Atherosclerosis	0.87	0.47 – 1.62	0.656
Chronic kidney disease	1.14	0.54 – 2.38	0.732
Stroke and/or CVD before PHF	0.86	0.39 – 1.89	0.712
Omarthrosis	1.67	0.14 – 19.88	0.685
Frozen shoulder	0.82	0.23 – 2.95	0.755
Analgesics*	0.93	0.52 – 1.65	0.802
Antibiotics*	0.46	0.07 – 2.89	0.406

*only within 14 days before admission. CVD – cerebrovascular disease, LPF – locked plate fixation, OR – odds ratio, PHF – proximal humeral fracture, RTSA – reverse total shoulder arthroplasty.

Table D9: Results from Cox regression model – overall survival.

Variable	HR	95% CI	p-value (two-sided)
Treatment group (RTSA vs LPF)	1.07	0.98 – 1.17	0.118
LOS during index (days)	1.02	1.01 – 1.03	$<$0.001
Male sex	1.56	1.00 – 2.45	0.052
Age at index surgery (years)	1.13	0.98 – 1.30	0.083
Year of index surgery (years)	1.10	0.70 – 1.73	0.679
Any anticoagulant	1.13	0.98 – 1.31	0.095
Any anti-osteoporotic drug	0.91	0.71 – 1.15	0.411
Osteoporosis	n.a.	n.a.	n.a.
Cancer	1.07	0.92 – 1.24	0.373
Diabetes Mellitus	1.51	0.99 – 2.28	0.053
Dementia	1.85	1.40 – 2.46	$<$0.001
Chronic polyarthritis	1.17	0.89 – 1.53	0.253
Obesity	0.96	0.47 – 1.96	0.905
Nicotine abuse	1.80	1.39 – 2.35	$<$0.001
Parkinson disease	1.52	1.05 – 2.19	0.026
Rotator cuff rupture	1.39	0.45 – 4.33	0.573
Alcohol abuse	2.18	1.00 – 4.77	0.051
Atrial fibrillation/ flutter	1.17	0.91 – 1.51	0.215
Hypertension	1.03	0.65 – 1.65	0.892
Congestive heart failure	1.45	1.11 – 1.89	0.007
Coronary heart disease	0.91	0.76 – 1.09	0.313
Atherosclerosis	0.94	0.79 – 1.11	0.444
Chronic kidney disease	1.40	1.15 – 1.70	$<$0.001
Stroke and/or CVD before PHF	1.00	0.82 – 1.23	0.981
Omarthrosis	1.18	0.64 – 2.17	0.593
Frozen shoulder	0.83	0.60 – 1.15	0.268
General compl. during IH	1.67	1.39 – 2.02	$<$0.001
Surgical compl. during IH	1.05	0.85 – 1.31	0.652
Start of COVID-19 pandemic	0.96	0.78 – 1.17	0.678
COVID-19 infection	2.23	1.76 – 2.84	$<$0.001

Start of COVID-19 pandemic and COVID-19 infection during follow-up were considered using time-dependent variables. CVD – cerebrovascular disease, HR – hazard ratio, IH – index hospitalization, LOS – length of hospital stay, LPF – locked plate fixation, PHF – proximal humeral fracture, RTSA – reverse total shoulder arthroplasty.

Table D10: Results from Cox regression model – major adverse events.

**Variable**	**HR**	**95% CI**	**p-value (two-sided)**
Treatment group (RTSA vs LPF)	1.04	0.97 – 1.12	0.270
LOS during index (days)	1.02	1.01 – 1.03	<0.001
Male sex	1.58	1.06 – 2.36	0.024
Age at index surgery (years)	1.06	0.93 – 1.20	0.383
Year of index surgery (years)	0.97	0.64 – 1.45	0.865
Any anticoagulant	1.01	0.89 – 1.15	0.856
Any anti-osteoporotic drug	0.94	0.76 – 1.16	0.552
Osteoporosis	n.a.	n.a.	n.a.
Cancer	1.06	0.94 – 1.21	0.331
Diabetes Mellitus	1.24	0.86 – 1.79	0.254
Dementia	1.71	1.33 – 2.20	<0.001
Chronic polyarthritis	1.25	0.99 – 1.57	0.061
Obesity	0.89	0.47 – 1.75	0.719
Nicotine abuse	1.41	1.13 – 2.35	0.002
Parkinson disease	1.51	1.09 – 2.08	0.014
Rotator cuff rupture	1.04	0.38 – 2.84	0.946
Alcohol abuse	1.72	0.86 – 3.45	0.127
Atrial fibrillation/ flutter	1.31	1.05 – 1.64	0.016
Hypertension	1.00	0.66 – 1.52	0.987
Congestive heart failure	1.23	0.97 – 1.56	0.087
Coronary heart disease	1.06	0.90 – 1.23	0.499
Atherosclerosis	0.96	0.83 – 1.11	0.571
Chronic kidney disease	1.23	1.17 – 1.64	<0.001
Stroke and/or CVD before PHF	1.11	0.93 – 1.33	0.238
Omarthrosis	0.94	0.55 – 2.17	0.821
Frozen shoulder	0.84	0.60 – 1.60	0.209
General compl. during IH	1.42	1.21 – 1.67	<0.001
Surgical compl. during IH	1.17	0.97 – 1.41	0.111
Start of COVID-19 pandemic	0.87	0.74 – 1.03	0.106
COVID-19 infection	2.16	1.72 – 2.73	<0.001

Start of COVID-19 pandemic and COVID-19 infection during follow-up were considered using time-dependent variables. CVD – cerebrovascular disease, HR – hazard ratio, IH – index hospitalization, LOS – length of hospital stay, LPF – locked plate fixation, PHF – proximal humeral fracture, RTSA – reverse total shoulder arthroplasty.

Table D11: Results from Cox regression model – thrombo-embolic events.

Variable	HR	95% CI	p-value (two-sided)
Treatment group (RTSA vs LPF)	1.09	1.00 – 1.17	0.039
LOS during index (days)	1.02	1.01 – 1.03	<0.001
Male sex	1.82	1.20 – 2.76	0.005
Age at index surgery (years)	1.05	0.93 – 1.20	0.424
Year of index surgery (years)	0.91	0.60 – 1.39	0.675
Any anticoagulant	1.00	0.87 – 1.14	0.975
Any anti-osteoporotic drug	0.95	0.76 – 1.18	0.643
Osteoporosis	n.a.	n.a.	n.a.
Cancer	1.12	0.98 – 1.28	0.095
Diabetes Mellitus	1.15	0.78 – 1.68	0.477
Dementia	1.82	1.40 – 2.37	<0.001
Chronic polyarthritis	1.06	0.83 – 1.36	0.625
Obesity	0.78	0.40 – 1.51	0.457
Nicotine abuse	1.48	1.17 – 1.87	0.001
Parkinson disease	1.45	1.03 – 2.04	0.035
Rotator cuff rupture	0.89	0.31 – 2.56	0.833
Alcohol abuse	1.50	0.72 – 3.09	0.277
Atrial fibrillation/ flutter	1.09	0.87 – 1.38	0.459
Hypertension	0.91	0.59 – 1.41	0.681
Congestive heart failure	1.22	0.95 – 1.56	0.120
Coronary heart disease	1.01	0.86 – 1.20	0.867
Atherosclerosis	0.97	0.83 – 1.13	0.655
Chronic kidney disease	1.31	1.10 – 1.57	0.002
Stroke and/or CVD before PHF	1.13	0.94 – 1.37	0.192
Omarthrosis	0.89	0.51 – 1.55	0.689
Frozen shoulder	0.87	0.64 – 1.17	0.343
General compl. during IH	1.60	1.34 – 1.90	<0.001
Surgical compl. during IH	1.16	0.95 – 1.41	0.143
Start of COVID-19 pandemic	0.92	0.77 – 1.10	0.400
COVID-19 infection	2.00	1.59 – 2.53	<0.001

Start of COVID-19 pandemic and COVID-19 infection during follow-up were considered using time-dependent variables. CVD – cerebrovascular disease, HR – hazard ratio, IH – index hospitalization, LOS – length of hospital stay, LPF – locked plate fixation, PHF – proximal humeral fracture, RTSA – reverse total shoulder arthroplasty.

Table D12: Results from Cox regression model – surgical complications.

Variable	HR	95% CI	p-value (two-sided)
Treatment group (RTSA vs LPF)	0.42	0.38 – 0.46	0<0.001
LOS during index (days)	1.01	1.01 – 1.02	0.001
Male sex	1.82	1.04 – 3.18	0.037
Age at index surgery (years)	0.95	0.80 – 1.13	0.570
Year of index surgery (years)	0.91	0.52 – 1.60	0.736
Any anticoagulant	1.08	0.92 – 1.27	0.333
Any anti-osteoporotic drug	0.98	0.73 – 1.30	0.875
Osteoporosis	n.a.	n.a.	n.a.
Cancer	1.05	0.90 – 1.24	0.519
Diabetes Mellitus	1.01	0.62 – 1.67	0.961
Dementia	1.21	0.86 – 1.70	0.276
Chronic polyarthritis	1.23	0.91 – 1.66	0.185
Obesity	1.14	0.47 – 2.78	0.776
Nicotine abuse	0.90	0.69 – 1.16	0.397
Parkinson disease	1.22	0.78 – 1.91	0.386
Rotator cuff rupture	0.90	0.22 – 3.67	0.885
Alcohol abuse	0.95	0.36 – 2.54	0.925
Atrial fibrillation/ flutter	0.97	0.71 – 1.32	0.837
Hypertension	1.14	0.64 – 2.02	0.654
Congestive heart failure	1.07	0.78 – 1.45	0.691
Coronary heart disease	0.99	0.81 – 1.22	0.937
Atherosclerosis	1.17	0.97 – 1.42	0.109
Chronic kidney disease	1.15	0.92 – 1.44	0.224
Stroke and/or CVD before PHF	1.04	0.82 – 1.32	0.762
Omarthrosis	0.86	0.43 – 1.70	0.662
Frozen shoulder	1.50	1.05 – 2.15	0.026
General compl. during IH	0.86	0.70 – 1.06	0.156
Surgical compl. during IH	1.22	0.98 – 1.51	0.070
Start of COVID-19 pandemic	0.80	0.65 – 0.98	0.031
COVID-19 infection	1.37	0.85 – 2.22	0.194

Start of COVID-19 pandemic and COVID-19 infection during follow-up were considered using time-dependent variables. CVD – cerebrovascular disease, HR – hazard ratio, IH – index hospitalization, LOS – length of hospital stay, LPF – locked plate fixation, PHF – proximal humeral fracture, RTSA – reverse total shoulder arthroplasty.

Table D13: Results from Cox regression model – minor outpatient complications.

Variable	HR	95% CI	p-value (two-sided)
Treatment group (RTSA vs LPF)	1.00	0.95 – 1.05	0.984
LOS during index (days)	1.00	0.99 – 1.00	0.310
Male Sex	1.07	0.94 – 1.21	0.327
Age at index surgery (years)	0.98	0.96 – 1.01	0.166
Year of index surgery (years)	1.02	0.93 – 1.11	0.720
Any anticoagulant	0.91	0.83 – 1.00	0.052
Any anti-osteoporotic drug	1.08	0.96 – 1.20	0.200
Osteoporosis	n.a.	n.a.	n.a.
Cancer	1.06	0.98 – 1.15	0.162
Diabetes Mellitus	0.89	0.80 – 0.99	0.031
Dementia	0.69	0.61 – 0.79	<0.001
Dementia	1.18	1.03 – 1.35	0.014
Obesity	1.23	1.05 – 1.43	0.010
Nicotine abuse	0.95	0.83 – 1.09	0.467
Rotator cuff rupture	1.87	1.43 – 2.43	<0.001
Atrial fibrillation/ flutter	1.05	0.94 – 1.17	0.435
Hypertension	1.18	1.02 – 1.36	0.028
Congestive heart failure	1.09	0.98 – 1.20	0.101
Coronary heart disease	1.03	0.94 – 1.13	0.518
Atherosclerosis	0.96	0.88 – 1.06	0.447
Chronic kidney disease	1.02	0.94 – 1.12	0.628
Stroke and/or CVD before PHF	1.05	0.96 – 1.14	0.271
Omarthrosis	1.88	1.54 – 2.30	<0.001
Frozen shoulder	4.15	3.43 – 5.03	<0.001
General compl. during IH	0.78	0.69 – 0.89	<0.001
Surgical compl. during IH	1.14	1.00 – 1.30	0.046
Start of COVID-19 pandemic	1.02	0.91 – 1.14	0.702
COVID-19 infection	1.08	0.83 – 1.40	0.557

Start of COVID-19 pandemic and COVID-19 infection during follow-up were considered using time-dependent variables. CVD – cerebrovascular disease, HR – hazard ratio, IH – index hospitalization, LOS – length of hospital stay, LPF – locked plate fixation, PHF – proximal humeral fracture, RTSA – reverse total shoulder arthroplasty.

Appendix E Sensitivity analysis – Different approaches for COVID-19.

Table E14: Sensitivity analysis considering COVID-19 – overall survival.

Variable	HR	95% CI	p-value (two-sided)
		**Approach 1**	
Treatment group (RTSA vs LPF)	1.04	0.98 – 1.10	0.235
LOS during index (days)	1.01	1.01 – 1.02	<0.001
Male sex	1.56	1.43 – 1.71	<0.001
Age at index surgery (years)	1.09	1.08 – 1.09	<0.001
Year of index surgery (years)	0.89	0.89 – 0.90	<0.001
Any anticoagulant	1.10	1.02 – 1.18	0.011
Any anti-osteoporotic drug	1.08	0.99 – 1.19	0.081
Osteoporosis	1.00	0.94 – 1.07	0.924
Cancer	1.06	0.99 – 1.13	0.087
Diabetes Mellitus	1.18	1.11 – 1.26	<0.001
Dementia	1.69	1.56 – 1.84	<0.001
Chronic polyarthritis	1.10	0.98 – 1.24	0.103
Obesity	0.87	0.81 – 0.94	<0.001
Nicotine abuse	1.36	1.18 – 1.56	<0.001
Parkinson disease	1.37	1.20 – 1.57	<0.001
Rotator cuff rupture	0.90	0.79 – 1.03	0.119
Alcohol abuse	1.51	1.31 – 1.74	<0.001
Atrial fibrillation/ flutter	1.19	1.10 – 1.29	<0.001
Hypertension	0.95	0.86 – 1.06	0.365
Congestive heart failure	1.25	1.17 – 1.34	<0.001
Coronary heart disease	0.98	0.92 – 1.05	0.636
Atherosclerosis	0.99	0.91 – 1.07	0.705
Chronic kidney disease	1.22	1.14 – 1.30	<0.001
Stroke and/or CVD before PHF	1.05	0.98 – 1.12	0.163
Omarthrosis	0.98	0.82 – 1.18	0.867
Frozen shoulder	0.90	0.78 – 1.04	0.150
General compl. during IH	1.50	1.37 – 1.64	<0.001
Surgical compl. during IH	1.05	0.94 – 1.18	0.407
		**Approach 2**	
Treatment group (RTSA vs LPF)	1.00	0.92 – 1.08	0.968
LOS during index (days)	1.01	1.01 – 1.02	<0.001
Male sex	1.50	1.34 – 1.68	<0.001
Age at index surgery (years)	1.09	1.09 – 1.10	<0.001
Year of index surgery (years)	0.98	0.96 – 1.01	0.134
Any anticoagulant	1.12	1.01 – 1.23	0.025
Any anti-osteoporotic drug	1.12	0.99 – 1.27	0.066
Osteoporosis	1.07	0.98 – 1.16	0.161
Cancer	1.04	0.95 – 1.13	0.410
Diabetes Mellitus	1.19	1.09 – 1.30	<0.001
Dementia	1.91	1.73 – 2.11	<0.001
Chronic polyarthritis	1.05	0.89 – 1.23	0.554
Obesity	0.80	0.72 – 0.89	<0.001
Nicotine abuse	1.49	1.25 – 1.79	<0.001
Parkinson disease	1.48	1.26 – 1.73	<0.001
Rotator cuff rupture	0.93	0.78 – 1.11	0.401
Alcohol abuse	1.50	1.27 – 1.78	<0.001
Atrial fibrillation/ flutter	1.20	1.08 – 1.32	<0.001
Hypertension	1.01	0.87 – 1.18	0.858
Congestive heart failure	1.31	1.20 – 1.44	<0.001
Coronary heart disease	0.96	0.88 – 1.05	0.400
Atherosclerosis	1.03	0.93 – 1.14	0.578
Chronic kidney disease	1.27	1.16 – 1.39	<0.001
Stroke and/or CVD before PHF	0.97	0.89 – 1.07	0.566
Omarthrosis	1.06	0.84 – 1.33	0.619
Frozen shoulder	0.95	0.78 – 1.15	0.572
General compl. during IH	1.53	1.37 – 1.72	<0.001
Surgical compl. during IH	1.16	1.01 – 1.33	0.033

Approach 1: COVID-19 infection was considered as a competing risk event.

Approach 2: Subset analysis including only patients with index surgery before 2020 ($N = 10,879$) and with end of follow-up defined as 31.12.2019.

CVD – cerebrovascular disease, HR – hazard ratio, IH – index hospitalization, LOS – length of hospital stay, LPF – locked plate fixation, PHF – proximal humeral fracture, RTSA – reverse total shoulder arthroplasty. Clustering of data (i.e. dependency of matching pairs) was not considered.

Table E15: Sensitivity analysis considering COVID-19 – major adverse events

Variable	HR	95% CI	p-value (two-sided)
**Approach 1**			
Treatment group (RTSA vs LPF)	1.04	0.99 – 1.10	0.138
LOS during index (days)	1.01	1.01 – 1.02	<0.001
Male sex	1.53	1.42 – 1.65	<0.001
Age at index surgery (years)	1.06	1.05 – 1.06	<0.001
Year of index surgery (years)	0.91	0.90 – 0.92	<0.001
Any anticoagulant	1.07	1.00 – 1.14	0.045
Any anti-osteoporotic drug	1.03	0.95 – 1.12	0.477
Osteoporosis	1.00	0.95 – 1.06	0.978
Cancer	1.05	0.99 – 1.11	0.102
Diabetes Mellitus	1.22	1.16 – 1.29	<0.001
Dementia	1.51	1.40 – 1.63	<0.001
Chronic polyarthritis	1.09	0.99 – 1.21	0.090
Obesity	0.93	0.87 – 0.99	0.020
Nicotine abuse	1.29	1.14 – 1.45	<0.001
Parkinson disease	1.32	1.16 – 1.49	<0.001
Rotator cuff rupture	0.97	0.87 – 1.08	0.578
Alcohol abuse	1.54	1.37 – 1.721	<0.001
Atrial fibrillation/ flutter	1.21	1.13 – 1.30	<0.001
Hypertension	1.06	0.97 – 1.16	0.229
Congestive heart failure	1.21	1.14 – 1.28	<0.001
Coronary heart disease	1.06	0.99 – 1.12	0.082
Atherosclerosis	1.00	0.94 – 1.07	0.910
Chronic kidney disease	1.29	1.22 – 1.37	<0.001
Stroke and/or CVD before PHF	1.09	1.03 – 1.16	0.004
Omarthrosis	0.99	0.86 – 1.15	0.92
Frozen shoulder	0.90	0.80 – 1.02	0.096
General compl. during IH	1.46	1.34 – 1.58	<0.001
Surgical compl. during IH	1.01	0.92 – 1.12	0.820
**Approach 2**			
Treatment group (RTSA vs LPF)	1.04	0.97 – 1.11	0.323
LOS during index (days)	1.01	1.01 – 1.02	<0.001
Male sex	1.48	1.34 – 1.63	<0.001
Age at index surgery (years)	1.06	1.06 – 1.07	<0.001
Year of index surgery (years)	1.00	0.98 – 1.01	0.623
Any anticoagulant	1.10	1.01 – 1.19	0.031
Any anti-osteoporotic drug	1.08	0.97 – 1.20	0.143
Osteoporosis	1.03	0.95 – 1.11	0.488
Cancer	1.02	0.95 – 1.10	0.605
Diabetes Mellitus	1.19	1.11 – 1.28	<0.001
Dementia	1.66	1.52 – 1.82	<0.001
Chronic polyarthritis	1.00	0.87 – 1.15	0.972
Obesity	0.86	0.79 – 0.94	<0.001
Nicotine abuse	1.42	1.23 – 1.65	<0.001
Parkinson disease	1.37	1.19 – 1.58	<0.001
Rotator cuff rupture	1.04	0.89 – 1.20	0.629
Alcohol abuse	1.52	1.31 – 1.75	<0.001
Atrial fibrillation/ flutter	1.24	1.14 – 1.36	<0.001
Hypertension	1.11	0.98 – 1.26	0.111
Congestive heart failure	1.26	1.17 – 1.36	<0.001
Coronary heart disease	1.03	0.95 – 1.12	0.469
Atherosclerosis	1.07	0.98 – 1.17	0.119
Chronic kidney disease	1.36	1.26 – 1.47	<0.001
Stroke and/or CVD before PHF	1.04	0.96 – 1.12	0.312
Omarthrosis	1.07	0.88 – 1.30	0.521
Frozen shoulder	0.91	0.78 – 1.08	0.287
General compl. during IH	1.46	1.32 – 1.61	<0.001
Surgical compl. during IH	1.13	1.00 – 1.27	0.048

Approach 1: COVID-19 infection was considered as a competing risk event.

Approach 2: Sensitivity analysis; Subset analysis including only patients with index surgery before 2020 ($N = 10,879$) and with end of follow-up defined as 31.12.2019.

CVD – cerebrovascular disease, HR – hazard ratio, IH – index hospitalization, LOS – length of hospital stay, LPF – locked plate fixation, PHF – proximal humeral fracture, RTSA – reverse total shoulder arthroplasty. Clustering of data (i.e. dependency of matching pairs) was not considered.

Table E16: Sensitivity analysis considering COVID-19 – thrombo-embolic events

Variable	HR	95% CI	p-value (two-sided)
	**Approach 1**		
Treatment group (RTSA vs LPF)	1.05	0.99 – 1.11	0.102
LOS during index (days)	1.01	1.01 – 1.02	<0.001
Male sex	1.51	1.39 – 1.64	<0.001
Age at index surgery (years)	1.07	1.07 – 1.08	<0.001
Year of index surgery (years)	0.90	0.89 – 0.91	<0.001
Any anticoagulant	1.06	0.99 – 1.13	0.105
Any anti-osteoporotic drug	1.06	0.97 – 1.15	0.202
Osteoporosis	1.02	0.96 – 1.09	0.452
Cancer	1.07	1.01 – 1.14	0.029
Diabetes Mellitus	1.17	1.10 – 1.24	<0.001
Dementia	1.59	1.47 – 1.72	<0.001
Chronic polyarthritis	1.06	0.95 – 1.19	0.273
Obesity	0.90	0.84 – 0.96	0.003
Nicotine abuse	1.27	1.12 – 1.44	<0.001
Parkinson disease	1.30	1.14 – 1.47	<0.001
Rotator cuff rupture	0.94	0.83 – 1.06	0.292
Alcohol abuse	1.44	1.28 – 1.63	<0.001
Atrial fibrillation/ flutter	1.16	1.08 – 1.25	<0.001
Hypertension	1.00	0.91 – 1.10	0.987
Congestive heart failure	1.18	1.11 – 1.26	<0.001
Coronary heart disease	1.00	0.93 – 1.06	0.873
Atherosclerosis	1.02	0.95 – 1.09	0.618
Chronic kidney disease	1.19	1.12 – 1.27	<0.001
Stroke and/or CVD before PHF	1.09	1.03 – 1.16	0.005
Omarthrosis	1.03	0.88 – 1.21	0.730
Frozen shoulder	0.95	0.83 – 1.08	0.443
General compl. during IH	1.46	1.34 – 1.59	<0.001
Surgical compl. during IH	1.04	0.93 – 1.15	0.507
	**Approach 2**		
Treatment group (RTSA vs LPF)	1.05	0.97 – 1.13	0.245
LOS during index (days)	1.01	1.01 – 1.02	<0.001
Male sex	1.44	1.30 – 1.61	<0.001
Age at index surgery (years)	1.08	1.07 – 1.08	<0.001
Year of index surgery (years)	0.98	0.96 – 1.00	0.025
Any anticoagulant	1.06	0.97 – 1.16	0.177
Any anti-osteoporotic drug	1.11	0.99 – 1.24	0.081
Osteoporosis	1.05	0.97 – 1.14	0.225
Cancer	1.06	0.98 – 1.15	0.146
Diabetes Mellitus	1.15	1.06 – 1.24	<0.001
Dementia	1.72	1.57 – 1.89	<0.001
Chronic polyarthritis	1.04	0.90 – 1.21	0.563
Obesity	0.86	0.78 – 0.94	0.001
Nicotine abuse	1.39	1.18 – 1.64	<0.001
Parkinson disease	1.35	1.16 – 1.56	<0.001
Rotator cuff rupture	0.96	0.82 – 1.13	0.602
Alcohol abuse	1.45	1.24 – 1.70	<0.001
Atrial fibrillation/ flutter	1.17	1.06 – 1.28	0.001
Hypertension	1.05	0.92 – 1.20	0.483
Congestive heart failure	1.26	1.16 – 1.37	<0.001
Coronary heart disease	0.98	0.90 – 1.06	0.554
Atherosclerosis	1.06	0.97 – 1.17	0.218
Chronic kidney disease	1.24	1.14 – 1.34	<0.001
Stroke and/or CVD before PHF	1.05	0.97 – 1.14	0.229
Omarthrosis	1.07	0.87 – 1.32	0.541
Frozen shoulder	0.95	0.80 – 1.13	0.556
General compl. during IH	1.50	1.35 – 1.67	<0.001
Surgical compl. during IH	1.15	1.02 – 1.31	0.029

Approach 1: COVID-19 infection was considered as a competing risk event.

Approach 2: Subset analysis including only patients with index surgery before 2020 ($N = 10,879$) and with end of follow-up defined as 31.12.2019.

CVD – cerebrovascular disease, HR – hazard ratio, IH – index hospitalization, LOS – length of hospital stay, LPF – locked plate fixation, PHF – proximal humeral fracture, RTSA – reverse total shoulder arthroplasty. Clustering of data (i.e. dependency of matching pairs) was not considered.

Table E17: Sensitivity analysis considering COVID-19 – surgical complications

Variable	HR	95% CI	p-value (two-sided)
**Approach 1**			
Treatment group (RTSA vs LPF)	0.47	0.42 – 0.52	<0.001
LOS during index (days)	1.01	1.00 – 1.01	0.054
Male sex	1.34	1.16 – 1.54	<0.001
Age at index surgery (years)	0.97	0.97 – 0.98	<0.001
Year of index surgery (years)	0.95	0.93 – 0.96	<0.001
Any anticoagulant	0.98	0.86 – 1.11	0.745
Any anti-osteoporotic drug	0.91	0.78 – 1.07	0.254
Osteoporosis	1.07	0.96 – 1.19	0.243
Cancer	1.04	0.93 – 1.16	0.472
Diabetes Mellitus	1.01	0.91 – 1.12	0.895
Dementia	1.02	0.86 – 1.20	0.859
Chronic polyarthritis	1.22	1.02 – 1.45	0.028
Obesity	1.23	1.09 – 1.37	<0.001
Nicotine abuse	1.08	0.90 – 1.30	0.415
Parkinson disease	1.28	1.01 – 1.61	0.039
Rotator cuff rupture	1.07	0.86 – 1.32	0.555
Alcohol abuse	1.23	1.02 – 1.49	0.030
Atrial fibrillation/ flutter	1.02	0.88 – 1.17	0.838
Hypertension	1.27	1.07 – 1.50	0.006
Congestive heart failure	1.02	0.90 – 1.56	0.736
Coronary heart disease	1.10	0.98 – 1.24	0.118
Atherosclerosis	1.06	0.94 – 1.21	0.343
Chronic kidney disease	1.13	1.00 – 1.26	0.043
Stroke and/or CVD before PHF	0.97	0.87 – 1.09	0.637
Omarthrosis	0.92	0.71 – 1.20	0.547
Frozen shoulder	1.18	0.96 – 1.46	0.122
General compl. during IH	1.08	0.90 – 1.29	0.412
Surgical compl. during IH	1.25	1.05 – 1.50	0.014
**Approach 2**			
Treatment group (RTSA vs LPF)	0.42	0.37 – 0.48	<0.001
LOS during index (days)	1.01	1.00 – 1.01	0.046
Male sex	1.38	1.16 – 1.65	<0.001
Age at index surgery (years)	0.97	0.96 – 0.98	<0.001
Year of index surgery (years)	0.98	0.95 – 1.00	0.088
Any anticoagulant	0.93	0.80 – 1.09	0.359
Any anti-osteoporotic drug	0.84	0.69 – 1.03	0.088
Osteoporosis	1.13	0.99 – 1.29	0.070
Cancer	1.04	0.91 – 1.19	0.601
Diabetes Mellitus	1.02	0.89 – 1.16	0.779
Dementia	1.14	0.94 – 1.38	0.196
Chronic polyarthritis	1.12	0.90 – 1.40	0.318
Obesity	1.19	1.03 – 1.37	0.016
Nicotine abuse	1.14	0.90 – 1.44	0.288
Parkinson disease	1.28	0.97 – 1.68	0.083
Rotator cuff rupture	1.22	0.96 – 1.56	0.111
Alcohol abuse	1.15	0.90 – 1.45	0.263
Atrial fibrillation/ flutter	1.05	0.89 – 1.25	0.572
Hypertension	1.26	1.02 – 1.56	0.031
Congestive heart failure	0.99	0.85 – 1.15	0.876
Coronary heart disease	1.13	0.98 – 1.31	0.100
Atherosclerosis	1.05	0.90 – 1.23	0.551
Chronic kidney disease	1.17	1.02 – 1.35	0.030
Stroke and/or CVD before PHF	1.07	0.93 – 1.24	0.320
Omarthrosis	0.77	0.54 – 1.11	0.160
Frozen shoulder	1.13	0.87 – 1.47	0.373
General compl. during IH	1.15	0.93 – 1.43	0.209
Surgical compl. during IH	1.12	0.88 – 1.39	0.385

Approach 1: COVID-19 infection was considered as a competing risk event.

Approach 2: Subset analysis including only patients with index surgery before 2020 ($N = 10,879$) and with end of follow-up defined as 31.12.2019.

CVD – cerebrovascular disease, HR – hazard ratio, IH – index hospitalization, LOS – length of hospital stay, LPF – locked plate fixation, PHF – proximal humeral fracture, RTSA – reverse total shoulder arthroplasty. Clustering of data (i.e. dependency of matching pairs) was not considered.

Table E18: Sensitivity analysis considering COVID-19 – minor outpatient complications

Variable	HR	95% CI	p-value (two-sided)
	**Approach 1**		
Treatment group (RTSA vs LPF)	1.02	0.96 – 1.08	0.470
LOS during index (days)	1.00	0.99 – 1.00	0.091
Male sex	0.92	0.84 – 1.02	0.097
Age at index surgery (years)	0.98	0.97 – 0.98	<0.001
Year of index surgery (years)	0.98	0.97 – 0.99	<0.001
Any anticoagulant	0.98	0.91 – 1.05	0.513
Any anti-osteoporotic drug	1.04	0.96 – 1.14	0.353
Osteoporosis	1.12	1.05 – 1.19	<0.001
Cancer	1.04	0.97 – 1.10	0.279
Diabetes Mellitus	0.91	0.86 – 0.97	0.005
Dementia	0.66	0.59 – 0.74	<0.001
Chronic polyarthritis	1.10	0.99 – 1.23	0.082
Obesity	1.19	1.12 – 1.28	<0.001
Nicotine abuse	0.99	0.88 – 1.11	0.802
Parkinson disease	0.82	0.68 – 0.97	0.022
Rotator cuff rupture	1.66	1.48 – 1.87	<0.001
Alcohol abuse	0.93	0.82 – 1.06	0.286
Atrial fibrillation/ flutter	0.96	0.88 – 1.05	0.336
Hypertension	1.10	1.00 – 1.20	0.042
Congestive heart failure	0.96	0.89 – 1.04	0.320
Coronary heart disease	1.04	0.97 – 1.12	0.235
Atherosclerosis	1.05	0.97 – 1.13	0.254
Chronic kidney disease	1.03	0.96 – 1.11	0.364
Stroke and/or CVD before PHF	1.06	0.99 – 1.14	0.087
Omarthrosis	1.79	1.58 – 2.03	<0.001
Frozen shoulder	3.41	3.08 – 3.79	<0.001
General compl. during IH	0.77	0.68 – 0.87	<0.001
Surgical compl. during IH	1.29	1.16 – 1.44	<0.001
	**Approach 2**		
Treatment group (RTSA vs LPF)	1.04	0.97 – 1.12	0.246
LOS during index (days)	1.00	0.99 – 1.00	0.180
Male sex	0.95	0.84 – 1.07	0.371
Age at index surgery (years)	0.98	0.97 – 0.99	<0.001
Year of index surgery (years)	1.00	0.99 – 1.02	0.659
Any anticoagulant	1.01	0.92 – 1.10	0.918
Any anti-osteoporotic drug	1.00	0.89 – 1.12	0.936
Osteoporosis	1.11	1.02 – 1.20	0.012
Cancer	1.02	0.94 – 1.11	0.642
Diabetes Mellitus	0.92	0.85 – 1.00	0.055
Dementia	0.71	0.62 – 0.82	<0.001
Chronic polyarthritis	1.08	0.94 – 1.24	0.268
Obesity	1.22	1.12 – 1.33	<0.001
Nicotine abuse	1.01	0.87 – 1.17	0.896
Parkinson disease	0.79	0.64 – 0.99	0.036
Rotator cuff rupture	1.69	1.46 – 1.94	<0.001
Alcohol abuse	0.94	0.80 – 1.11	0.481
Atrial fibrillation/ flutter	0.94	0.84 – 1.05	0.261
Hypertension	1.06	0.94 – 1.19	0.325
Congestive heart failure	1.02	0.93 – 1.12	0.715
Coronary heart disease	1.02	0.93 – 1.12	0.684
Atherosclerosis	1.00	0.91 – 1.11	0.979
Chronic kidney disease	1.01	0.92 – 1.10	0.885
Stroke and/or CVD before PHF	1.04	0.95 – 1.13	0.432
Omarthrosis	1.83	1.57 – 2.14	<0.001
Frozen shoulder	3.59	3.17 – 4.06	<0.001
General compl. during IH	0.79	0.68 – 0.92	0.002
Surgical compl. during IH	1.25	1.09 – 1.44	0.001

Approach 1: COVID-19 infection was considered as a competing risk event.

Approach 2: Subset analysis including only patients with index surgery before 2020 ($N = 10,879$) and with end of follow-up defined as 31.12.2019.

CVD – cerebrovascular disease, HR – hazard ratio, IH – index hospitalization, LOS – length of hospital stay, LPF – locked plate fixation, PHF – proximal humeral fracture, RTSA – reverse total shoulder arthroplasty. Clustering of data (i.e. dependency of matching pairs) was not considered.
